# Hypocretin‐1/Hypocretin Receptor 1 Regulates Neuroplasticity and Cognitive Function through Hippocampal Lactate Homeostasis in Depressed Model

**DOI:** 10.1002/advs.202405354

**Published:** 2024-08-09

**Authors:** Bing Chen, Kangyu Jin, Jingyi Dong, Shangping Cheng, Lingzhuo Kong, Shaohua Hu, Zuobing Chen, Jing Lu

**Affiliations:** ^1^ Department of Psychiatry the First Affiliated Hospital Zhejiang University School of Medicine Hangzhou 310003 China; ^2^ Zhejiang Key Laboratory of Precision psychiatry Hangzhou 310003 China; ^3^ Department of Rehabilitation Medicine The First Affiliated Hospital Zhejiang University School of Medicine Hangzhou 310003 China

**Keywords:** cognition, depression, hypocretin‐1, hypoxia‐inducible factor‐1α, lactate

## Abstract

Cognitive dysfunction is not only a common symptom of major depressive disorder, but also a more common residual symptom after antidepressant treatment and a risk factor for chronic and recurrent disease. The disruption of hypocretin regulation is known to be associated with depression, however, their exact correlation is remains to be elucidated. Hypocretin‐1 levels are increased in the plasma and hypothalamus from chronic unpredictable mild stress (CUMS) model mice. Excessive hypocretin‐1 conducted with hypocretin receptor 1 (HCRTR1) reduced lactate production and brain‐derived neurotrophic factor (BDNF) expression by hypoxia‐inducible factor‐1α (HIF‐1α), thus impairing adult hippocampal neuroplasticity, and cognitive impairment in CUMS model. Subsequently, it is found that HCRTR1 antagonists can reverse these changes. The direct effect of hypocretin‐1 on hippocampal lactate production and cognitive behavior is further confirmed by intraventricular injection of hypocretin‐1 and microPET‐CT in rats. In addition, these mechanisms are further validated in astrocytes and neurons in vitro. Moreover, these phenotypes and changes in molecules of lactate transport pathway can be duplicated by specifically knockdown of HCRTR1 in hippocampal astrocytes. In summary, the results provide molecular and functional insights for involvement of hypocretin‐1‐HCRTR1 in altered cognitive function in depression.

## Graphical abstract

This diagram illustrates the mechanism by which hypocretin‐1 contributes to cognitive impairment in depression. The study's findings demonstrate that elevated hypocretin‐1 suppress HIF‐1a expression in hippocampal astrocytes, resulting in decreased lactate production and release from astrocytes. This ultimately impairs neurogenesis and synaptic plasticity, thereby contributing to depressive‐like behavior and cognitive deficits.

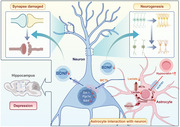

## 1 Introduction

Major depressive disorder (MDD) stands as one of the most prevalent psychiatric disorders worldwide.^[^ ^1^, ^2^ ^]^ The pathological features of this disease include the reduction of hippocampal volume and the destruction of neuroplasticity.^[^ ^3^, ^4^, ^5^ ^]^ Cognitive dysfunction is a ubiquitous symptom, persists in affecting the quality of life and social functioning of patients with MDD.^[^ ^6^ ^]^ Nonetheless, the intricate pathophysiological mechanisms driving cognitive dysfunction in MDD remain elusive.

Hypocretins act by binding to and activating GPCRs (hypocretin receptor 1 (HCRTR1) and hypocretin receptor 2 (HCRTR2)).^[^ ^7^ ^]^ Hypocretin‐1, a neuropeptide synthesized in the hypothalamus that preferentially binds to HCRTR1,^[^ ^8^ ^]^ plays a pivotal role in the regulation of vital physiological functions by projecting to key brain regions, including the prefrontal cortex, hippocampus, and locus coeruleus.^[^ ^9^ ^]^ Hypocretin‐1 and HCRTR1 are emerging as regulators of hippocampal neurogenesis and synaptic plasticity.^[^ ^10^, ^11^ ^]^ Our previous investigations have unveiled elevated expression of hypocretin‐1 in both postmortem hypothalamus samples and plasma collected from patients diagnosed with MDD.^[^ ^12^, ^13^, ^14^ ^]^ Furthermore, we have observed a correlation between hypocretin‐1 levels and cognitive function in individuals with MDD.^[^ ^15^ ^]^ Intriguingly, hypocretin‐1 has also been implicated in the pathogenesis of major neurocognitive disorders such as Alzheimer's disease.^[^ ^16^, ^17^, ^18^ ^]^ Experimental data from studies on 3xTg‐AD mice have indicated that hypocretin‐1 exacerbates cognitive deficits and synaptic plasticity impairment,^[^ ^19^ ^]^ Additionally, a clinical report has documented enhanced cognitive performance in individuals with psychiatric disorders who switched from benzodiazepines to hypocretin receptor antagonists (e.g., Suvorexant and Lemborexant).^[^ ^20^ ^]^ Despite these findings, the precise mechanisms through which hypocretin‐1 is involved in cognitive impairment in depression are predominantly unexplored.

Glucose metabolism disorder plays a pivotal role in the pathogenesis of depression. Extensive evidence suggests that depressed patients and animal models exhibit impaired glucose metabolism in the hippocampus, intricately linked to cognitive function.^[^ ^21^, ^22^, ^23^, ^24^ ^]^ The hippocampus, a crucial brain region for regulating cognitive function, particularly learning and memory,^[^ ^25^ ^]^ there is a pressing need to delve into the mechanisms underlying cognitive impairment resulting from disturbed energy metabolism in the hippocampi of individuals with MDD. Lactate, a significant energy substrate derived from pyruvate through reverse catalysis during glycolysis,^[^ ^26^ ^]^ is indispensable maintaining neural functions and contributes to cognitive process.^[^ ^27^, ^28^ ^]^ Although the hypocretin system has been found to regulate peripheral blood glucose levels,^[^ ^29^ ^]^ and decrease lactate production by down‐regulating glucose transporter expression and lactate dehydrogenase A (LDHA) in HepG2 cells.^[^ ^30^ ^]^ There remains a dearth of understanding regarding how hypocretin‐1 regulates glucose metabolism and lactate production at the molecular level.

The objective of this study was to investigate the potential mechanism underlying hypocretin dysfunction and cognitive impairment in depression. In depression, hypocretin‐1 may impose an adverse regulatory effect on the hypoxia‐inducible factor‐1α (HIF‐1α) pathway through hypocretin receptor 1, thereby disrupting the glycolytic pathway and resulting in reduced lactate release from astrocytes. Consequently, downstream brain‐derived neurotrophic factor (BDNF) expression is diminished, ultimately impairing synaptic plasticity and neurogenesis of hippocampal neurons and contributing to cognitive deficits observed in depressed individuals.

## 2 Results

### 2.1 SB‐334867 Ameliorated Anxiety and Depressive‐Like Behaviors Induced by Chronic Unpredictable Mild Stress (CUMS), as well as Cognitive Impairment

**Figure 1:**
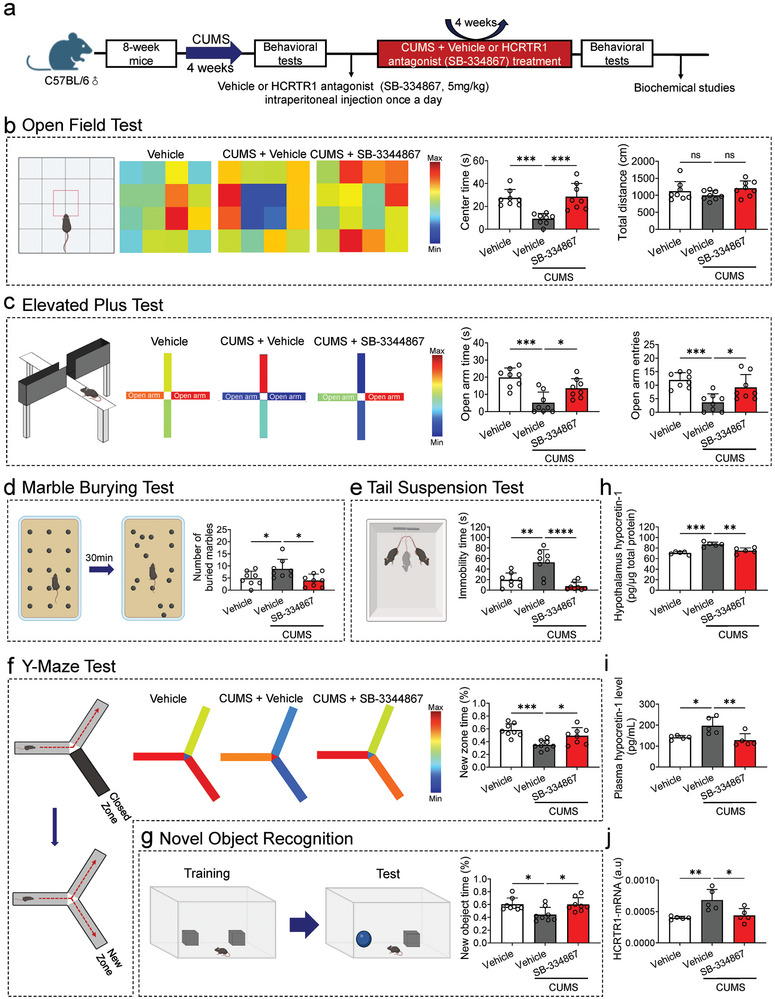
Alleviation effects of hypocretin receptor 1 (HCRTR1) antagonist (SB‐334867) on chronic unpredictable mild stress (CUMS)‐induced anxiety and depressive‐behaviors and cognitive repairment. a) Schematic illustration of the construction of CUMS‐induced depression model and the therapeutic treatment of HCRTR1 antagonist. Vehicle, control; CUMS, CUMS + Vehicle; SB334867, CUMS + SB334867. b) Diagram of open field test and typical heatmap of mice exploration during the open field test. SB‐334867 reversed CUMS‐induced decreasing time in the central zone, while there was no difference of total distance among three groups. c) Diagram of elevated plus test and heatmap of mice exploration during the elevated plus test. SB‐334867 reversed CUMS‐induced decreasing time and entries in open arms. d) Diagram of marble burying test. SB‐334867 reversed the CUMS‐induced increase in the number of buried marbles. e) Diagram of tail suspension test. SB‐334867 reversed the CUMS‐induced increased immobility time. (f) Diagram of Y‐maze test. SB‐334867 reversed the CUMS‐induced less time proportion in exploration in the new zone. g) Diagram of novel object recognition test. SB‐334867 reversed the CUMS‐induced less time proportion in exploration in the novel object. h,i) Hypothalamus and plasma hypocretin‐1 level in different groups. j) The mRNA expression of HCRTR1 in hippocampus in different groups. Dots in panels represent individual samples. Data were shown as mean ± SD. One‐way ANOVA followed by Dunnett's post hoc test. *p < 0.05, **p < 0.01, ***p < 0.001, ****p < 0.0001; ns, no significant difference, *p *≥ 0.05.

The CUMS model was employed to induce depressive‐like phenotypes in mice (**Figure** **1**a). CUMS mice exhibited significant anxiety and depressive‐like behaviors in the open field test (OFT), elevated plus maze (EPM), marble burying test (MBT) and tail suspension test (TST), respectively, along with cognitive impairment in the Y‐maze and novel object recognition (NOR) tests, which were effectively reversed by an HCRTR1 antagonist (SB‐334867), Specifically, compared to the vehicle group (control), the CUMS group (CUMS + vehicle) displayed reduced exploration time in the central area; however, this effect was reversed following treatment with SB‐334867. There were no significant differences observed among the three groups regarding total distance traveled during testing sessions (Figure 1b). Furthermore, in the EPM test, the CUMS group showed shorter exploration time and reduced entries in the open arm, which was significantly improved in SB‐334867 group (CUMS + SB‐334867) (Figure 1c). Additionally, a lower number of buried marbles was observed in mice treated with SB‐334867 compared to those from the CUMS group during MBT assessment (Figure 1d). The increased immobility time seen in TST for mice subjected to CUMS was also attenuated after treatment with SB‐334867 (Figure 1e). Moreover, both Y‐maze and NOR tests revealed that cognitive impairment occurred within the CUMS group as indicated by a significant reduction in time spent exploring new zones or objects; however, these impairments were significantly alleviated following treatment with SB‐33486 (Figure 1f,g). In addition, behavioral tests were conducted on the control group following intraperitoneal injection of SB‐334867 over a 4‐week period to further validate the effects of SB‐334867 on control behaviors. Compared to the vehicle group, no significant differences were found in exploration time in the center of the open field test and open arm of the elevated plus maze, as well as in the proportion of new zone and novel object time in Y‐maze and NOR, respectively. Additionally, there were no significant differences in immobility time in the TST for mice treated with SB‐334867, suggesting that SB‐334867 itself has no effect on the behavioral performance of control mice (Figure S1, Supporting Information). We have previously reported elevated plasma hypocretin‐1 levels associated with depression patients' cognitive performance.^[^ ^15^ ^]^ In this study, we confirmed that the hypocretin system was dysregulated in CUMS mice. Compared to the vehicle group, plasma and hypothalamic hypocretin‐1 levels were significantly increased in the CUMS group, as well as the mRNA level of HCRTR1 in the hippocampus (Figure 1h–j). According to the above results, HCRTR1 antagonists can restore the hypocretin system imbalance and ameliorate the anxiety and depressive‐like behaviors and cognitive impairment induced by CUMS.

### 2.2 Effects of SB‐334867 on Synaptic Plasticity and Hippocampal Adult Neurogenesis

**Figure 2:**
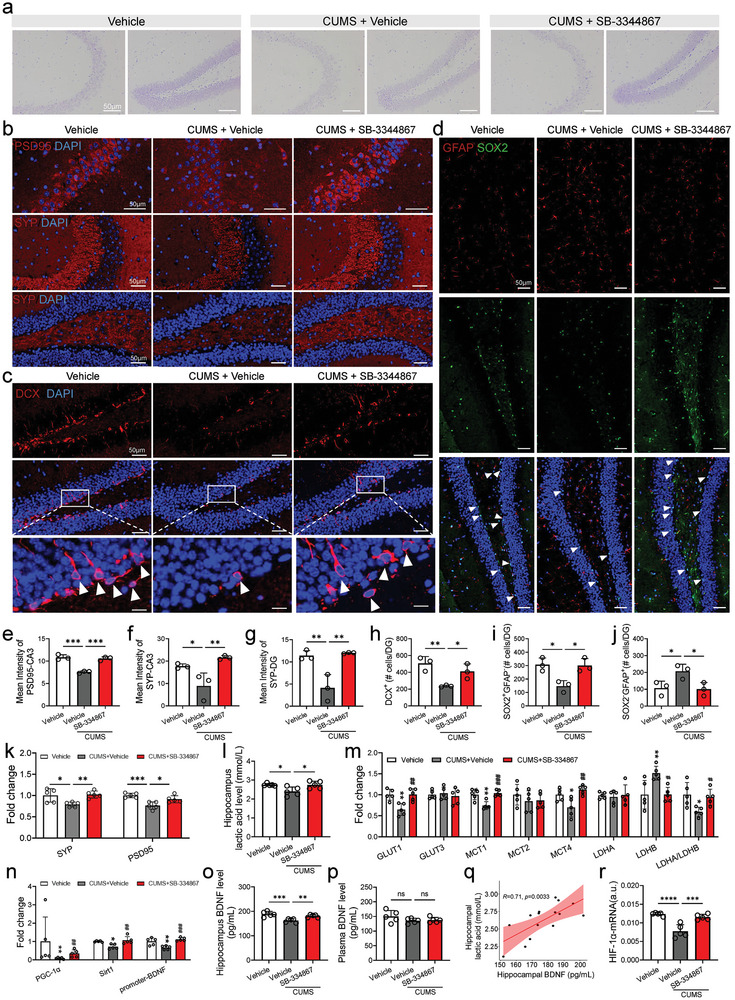
HCRTR1 antagonist (SB‐334867) restored hippocampal synaptic plasticity and neurogenic dysregulation induced by CUMS. a–d) Representative microscopic of Nissl staining, postsynaptic density protein 95 (PSD‐95) and synaptophysin (SYP), doublecortin (DCX) positive cell and GFAP/SOX2 positive cells within the hippocampus in different groups, respectively. scale bar, 50 µm. e–g) Quantification of mean fluorescence intensity of PSD‐95 and SYP. h) Quantification of DCX positive cells in DG. i,j) Quantification of SOX2^+^/GFAP^−^ and SOX2^−^/GFAP^+^ cells in DG, respectively. k) The mRNA expression of PSD‐95 and SYP in hippocampus. l) Hippocampal lactate level in different groups. m) Changes of mRNA expression of glycolytic‐related factors in hippocampus of different groups. GLUT, glucose transporter; MCT, monocarboxylic acid transporter; LDH, lactate dehydrogenase. *Compared with vehicle, #compared with CUMS. n) Changes of mRNA expression of proliferator‐activated receptor gamma coactivator alpha (PGC‐1α)‐ silent information regulator 1 (Sirt1)‐ brain‐derived neurotrophic factor (BDNF) in hippocampus of different groups. *Compared with vehicle, #compared with CUMS. o,p) Hippocampal and plasma BDNF level in different groups. q) The correlation between hippocampal BDNF and lactate levels. r) The mRNA expression of hypoxia‐inducible factor 1α (HIF‐1α) in hippocampus in different groups. Dots in panels represent individual samples. Data were shown as mean ± SD. One‐way ANOVA followed by Dunnett's post hoc test. *p < 0.05, **p < 0.01, ***p < 0.001, ****p < 0.0001; #*p* < 0.05, ##*p* < 0.01, ###*p* < 0.001, ####*p* < 0.0001; ns, no significant difference, *p *≥ 0.05.

Disruption of synaptic plasticity is regarded as be one of the neurobiological mechanisms underlying depression.^[^ ^31^ ^]^ The expression levels of synaptophysin (SYP) and postsynaptic densitin‐95 (PSD‐95), synaptic‐associated proteins in the hippocampus, serve as markers for evaluating synaptic integrity and plasticity.^[^ ^32^, ^33^ ^]^ Previous researches have consistently demonstrated a significant reduction in their expression levels among patients with MDD,^[^ ^34^, ^35^ ^]^ thus justifying their inclusion in this study. Our immunofluorescence analysis revealed a significant decrease in the mean fluorescence intensity of PSD‐95 in the CA3 region of the CUMS group compared to the vehicle group (**Figure** **2**b,e), while SYP expression was significantly reduced in both the CA3 and DG regions, which was ameliorated by SB‐334867 treatment (Figure 2b,f,g). Similarly, our qPCR results confirmed that treatment with SB‐334867 also rescued the CUMS‐induced the reduction in mRNA expression of neuroplasticity‐related markers (PSD‐95, SYP) in the hippocampus caused by CUMS (Figure 2k). Chronic stress disrupts local protein synthesis in synapses, leading to changes in the production of proteins required for synaptic formation, maturation, and function. These findings demonstrate that HCRTR1 antagonists ameliorate CUMS‐induced neuroplasticity dysregulation.

Neuronal dysfunction in the hippocampus might potentially contribute to depression.^[^ ^36^ ^]^ The Nissl staining was used to observe morphological changes in hippocampal neurons. Compared with the vehicle group, CUMS group showed more significantly damaged neurons with disintegrating Nissl vesicles and a reduction of Nissl‐positive cells, which was improved after treatment with SB‐334867 (Figure 2a; Figure S2, Supporting Information). Alterations in adult hippocampal neurogenesis have been implicated the development of stress‐induced psychological disorders, including depression, learning and memory.^[^ ^37^, ^38^ ^]^ Doublecortin (DCX) is a microtubule‐associated protein expressed almost exclusively in immature neurons. As a marker of neuronal precursor cells, it is thought to be involved in neuronal migration and development.^[^ ^39^ ^]^ The number of DCX^+^ cells exhibited a significant reduction in CUMS group, compared with the vehicle group, which was reversed in the SB‐334867 group (Figure 2c,h). New neurons are generated by neural stem cells (NSCs) in the adult hippocampus,^[^ ^40^ ^]^ of which development requires SOX2,^[^ ^41^ ^]^ representing NSCs that have the potential to produce both neurons and astrocytes.^[^ ^42^ ^]^ There was a significant increase in the number of SOX2^−^GFAP^+^ cells in DG of CUMS, while the number of SOX2^+^GFAP^−^ cells decreased, implying that decreasing cells have the potential to differentiate into neurons; however, the SB‐334867 reverse this change (Figure 2d,i,j). In conclusion, HCRTR1 antagonists could potentially mitigate depressive‐like behaviors and cognitive impairment by promoting neural progenitor cells and hippocampal adult neurogenesis.

### 2.3 Hypocretin‐1 Depend on HIF‐1α to Regulate Lactate Production and Dysregulation of Glycolysis in the Hippocampus, Leading to Neuroplasticity Damage

We next try to explore the underlying mechanism by which HCRTR1 antagonists affect neuroplasticity. As the preferred substrate for maintaining neuronal activity in the brain, lactate is essential for synaptic plasticity and memory,^[^ ^27^ ^]^ playing an important role in the development of depression.^[^ ^43^ ^]^ The decrease in hippocampal lactate levels in CUMS mice could be reversed by the treatment of SB‐334867 (Figure 2l). Lactate produced after glycogen breakdown and glycolysis in astrocytes is transported to neurons by monocarboxylic acid transporters (MCTs).^[^ ^44^, ^45^, ^46^ ^]^ The conversion between lactate and pyruvate is catalyzed by lactate dehydrogenases (LDHs), and the two subtypes LDHA and LDHB are responsible for the conversion of pyruvate to lactate and the reverse reaction, respectively.^[^ ^47^ ^]^ The mRNA expression of factors related to glycolysis, including glucose transporter 1 (GLUT1), MCT1 and MCT4, were significantly decreased, while the mRNA level of LDHB increased, leading to the ratio of LDHA/LDHB decreased significantly (Figure 2m). Thus, reduced glucose uptake and decreased LDHs activity are possible causes of decreased lactate levels and the decreased expression of MCT1 and MCT4 may lead to reduced lactate transport to neurons, causing synaptic damage and cognitive impairment. It has long been established that hippocampal BDNF is associated with depression and cognitive impairment.^[^ ^48^, ^49^ ^]^ Existing evidence suggest that lactate may increase BDNF levels, thereby exerting a neuroprotective effect and improving cognition, and lactate‐induced BNDF expression is achieved through silent information regulator 1 (Sirt1)‐dependent induction of peroxisome proliferator‐activated receptor gamma coactivator alpha (PGC‐1α).^[^ ^50^ ^]^ Consistent with it, our results reflected that hippocampal BDNF level was significantly reduced in CUMS mice, while mRNA expression of PGC‐1α, Sirt1, and promoter‐BDNF was also significantly decreased in CUMS group and increased after treatment (Figure 2o,p. Besides, the hippocampal BDNF and lactate exhibited a significant positive correlation (Figure 2q), suggesting that lactate may play a neuroprotective role and improve cognition through the PGC‐1α‐Sirt1‐BDNF pathway. It is well known that HIF‐1α regulates glucose metabolism, which mainly depends on the upregulation of genes for glucose transporter proteins (GLUT1 and GLUT3) and enzymes of the glycolytic pathway.^[^ ^51^, ^52^ ^]^ In accordance with this, the mRNA expression of HIF‐1α were significantly down‐regulated in the hippocampus of CUMS mice, which recovered after treatment with SB‐334867 (Figure 2r). Hence, elevated hypocretin‐1 level may regulate the expression of glycolysis‐related factors and lactate release in the hippocampus through negative regulation of HIF‐1α, contributing to impaired neuroplasticity and cognitive impairment in depression.

### 2.4 Directly Elevated Hypocretin‐1 can Cause Depressive‐Like Behavior and Cognitive Impairment, Affect Hippocampal Glucose Metabolism

**Figure 3:**
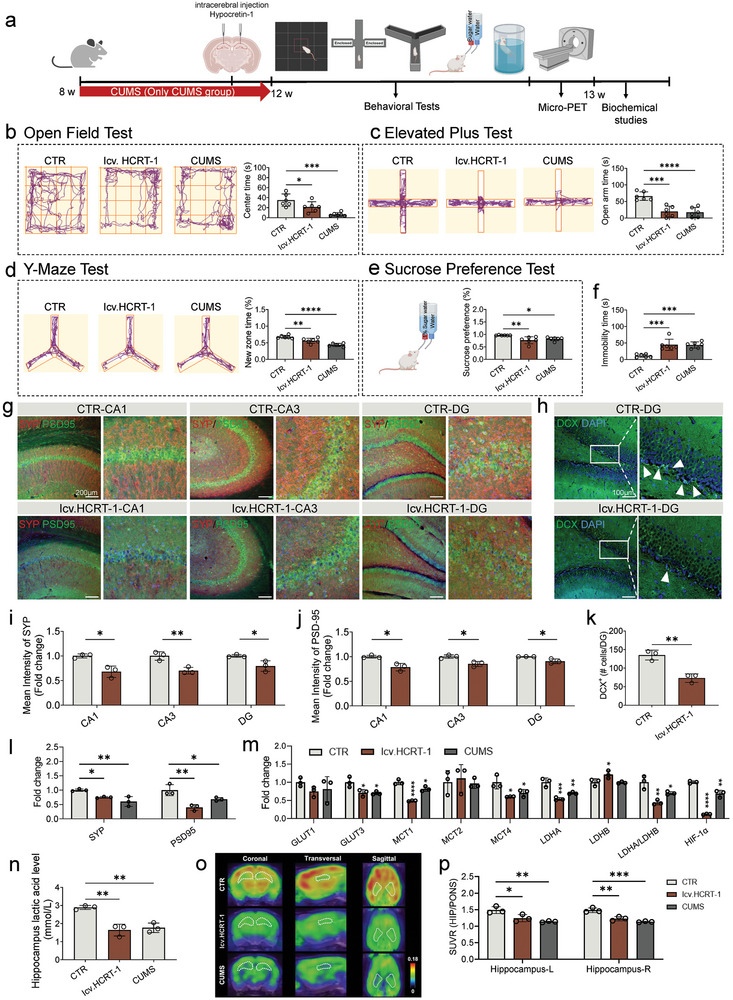
Hypocretin‐1 induced anxiety, depressive‐behaviors, cognitive impairment and dysregulation of energy metabolism. a) Schematic illustration of the intraventricular injection hypocretin‐1 (icv.HCRT‐1) in hippocampus and construction of CUMS‐induced depression model. b) Both icv.HCRT‐1 and CUMS resulted in decreased center time in open field test. c) Both icv.HCRT‐1 and CUMS decreased open arm time in elevated plus test. d) Both icv.HCRT‐1 and CUMS resulted in decreased new zone exploration time in Y‐maze test. e) Both icv.HCRT‐1 and CUMS resulted in decreased sucrose preference. f) Both icv.HCRT‐1 and CUMS resulted in increased immobility time in forced swim test. g) Representative microscopic of PSD‐95 and SYP in hippocampus. scale bar, 200 µm. h) Representative microscopic of DCX positive cells in the DG. scale bar, 100 µm. i,j) Quantification of mean fluorescence intensity SYP and PSD‐95, respectively. k) Quantification of DCX positive cells in the DG. l) mRNA expression of synapse‐related gene (SYP and PSD‐95) in hippocampus. m) Changes of mRNA expression of glycolytic‐related factors in hippocampus of different groups. n) Hippocampal lactate level in different groups. o) Representative ^18^F‐FDG PET images of rat hippocampus from control (CTR), icv.HCRT‐1 and CUMS groups. p) Glucose metabolism in hippocampus. Dots in panels represent individual samples. Data were shown as mean ± SD. One‐way ANOVA followed by Dunnett's post hoc test. *p < 0.05, **p < 0.01, ***p < 0.001, ****p < 0.0001; ns, no significant difference, *p *≥ 0.05.

To ascertain the specific role of hypocretin‐1, it was directly administered via intracerebroventricular injection (**Figure** **3**a). Similar with CUMS, intraventricular injection hypocretin‐1 (icv.HCRT‐1) rats exhibited anxiety and depressive‐like behaviors and impaired cognition. Specifically, compared with the CTR group, both icv.HCRT‐1 and CUMS rats displayed shorter exploration time in the center area in OFT and time in the open arm in EPM, respectively (Figure 3b,c). In sucrose preference test (SPT) revealed reduced sucrose preference in icv‐HCRT‐1 and CUMS rats (Figure 3e). Correspondingly, both icv.HCRT‐1 and CUMS had prolonged the immobility time in the FST (Figure 3f). In Y‐maze test, icv.HCRT‐1 and CUMS rats demonstrated cognitive impairment, as indicated by a significant decrease in the proportion of time spent exploring the new arm compared to the CTR group (Figure 3d). These investigations suggest that elevated level of hypocretin‐1 can cause anxiety and depressive‐like behaviors and cognitive impairment. Furthermore, in accordance with previous CUMS‐induced synaptic damage, the results further illustrated that the expression of synapse‐associated proteins (SYP and PSD‐95) of the hippocampus (among CA1, CA3, and DG regions) was significantly reduced in the icv.HCRT‐1 group compared with the CTR group (Figure 3g–j), consistent with mRNA expression (Figure 3l), reflecting that elevated hypocretin‐1 may contribute to synapse damage. Additionally, the number of DCX^+^ cells was significantly decreased in icv.HCRT‐1 (Figure 3h,k). These imply that the icv.HCRT‐1‐induced behavioral impairment may also be due to synaptic and neurogenic damage.

Otherwise, coincident with the decrease of lactate induced by CUMS, the lactate level in the hippocampus of icv.HCRT‐1 rats also exhibited a significant decrease (Figure 3n). Lactate is made primarily from glucose.^[^ ^53^ ^]^ Central glucose metabolism disorder plays an important role in the pathogenesis of depression, and glucose metabolic homeostasis may be closely related to cognitive function.^[^ ^54^, ^55^ ^]^ To investigate the effect of hypocretin‐1 on hippocampal glucose metabolism, ^18^Fluorodeoxyglucose  PET/CT scans were performed on icv.HCRT‐1 rats. icv.HCRT‐1 significantly reduced the uptake of ^18^F‐FDG, which was consistent with CUMS group (Figure 3o,p). The mRNA expression of factors related to glycolysis were also disordered in icv.HCRT‐1 rats, with significantly reduced expression of GLUT3, MCT1, 4 and LDHA, and increased LDHB expression (Figure 3m). At the same time, the expression of HIF‐1α in the hippocampus of icv.HCRT‐1 rats were significantly decreased (Figure 3m). Altogether, these findings suggest that elevated hypocretin‐1 levels may induce impaired glycolysis through the regulation of HIF‐1α, culminating in decreased lactate, synaptic damage, and cognitive impairment.

### 2.5 Hypocretin‐1 Target Reduced Lactate Transmission from Hippocampal Astrocytes to Neurons

**Figure 4:**
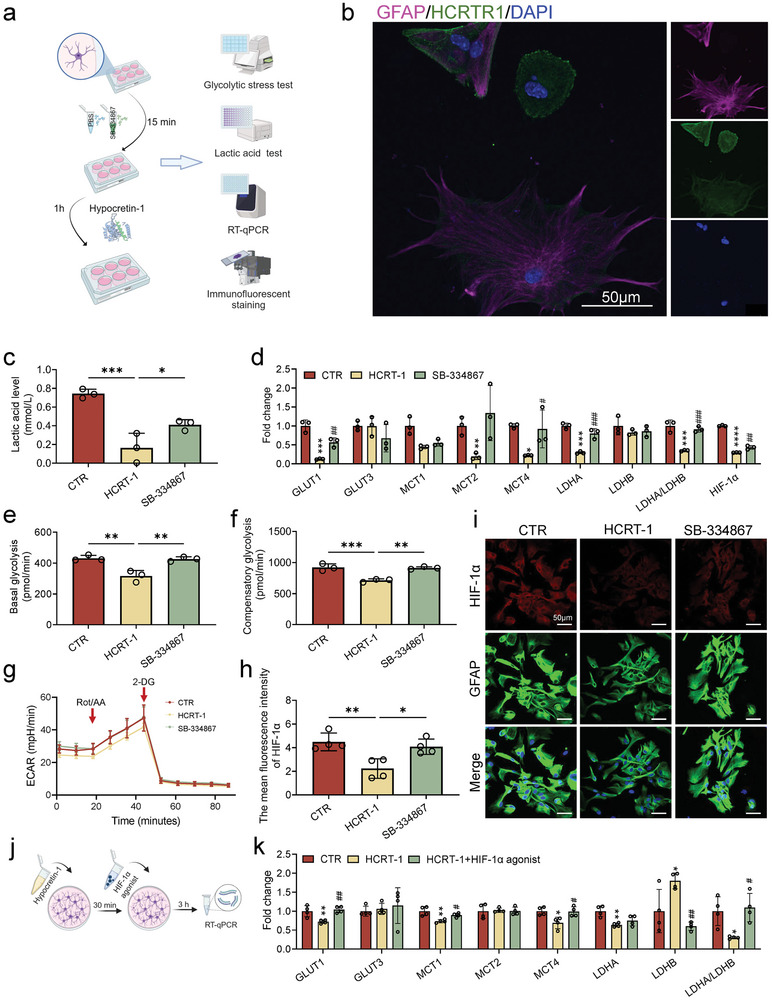
Hypocretin‐1 induced the decrease of lactate and glycolysis rate in hippocampal astrocytes. a) Schematic diagram showing the detection of glycolytic analysis, lactate, RT‐qPCR and immunofluorescent staining. SB‐334867, a HCRTR1 antagonist. b) The co‐expression of HCRTR1 and GFAP. c) The lactate in astrocyte medium in different groups. CTR, control; HCRT‐1, hypocretin‐1; SB‐334867, HCRT‐1 + SB‐334867. d) Changes of mRNA expression of glycolytic‐related factors in astrocytes. *Compared with CTR, #compared with HCRT‐1. e–g) Glycolysis rate test. The basal, compensatory glycolysis rate and extracellular acidification rate (ECAR) of astrocytes, respectively. h) Quantification of mean fluorescence intensity of the HIF‐1α expression in astrocytes. i) Representative microscopic images showing expression of HIF‐1α in astrocytes. j) Schematic diagram showing treatment with HIF‐1α agonist. k) Changes of mRNA expression of glycolytic‐related factors in astrocytes treatment with HCRT‐1 and HIF‐1α agonist. Dots in panels represent individual samples. Data were shown as mean ± SD. One‐way ANOVA followed by Dunnett's post hoc test. *p < 0.05, **p < 0.01, ***p < 0.001, ****p < 0.0001; #*p* < 0.05, ##*p* < 0.01, ###*p* < 0.001, ####*p* < 0.0001; ns, no significant difference, *p *≥ 0.05.

Subsequently, we performed in vitro experiments on hippocampal astrocytes to investigate the possible mechanisms by which hypocretin‐1 induces decreased lactate, synaptic damage and cognitive impairment (**Figure** **4**a). Astrocytes, the predominant cell population in the brain, play a direct role in the pathophysiology of depression, providing nutritional support, contributing to the maintenance of synaptic plasticity and regulating brain homeostasis.^[^ ^56^ ^]^ The expression of HCRTR1 in astrocytes was validated (Figure 4b). Following the administration of exogenous hypocretin‐1, a significant reduction of lactate level in the medium was observed, while it was improved in the groups pre‐treated with SB‐334867 (Figure 4c). To determine whether the decrease in lactate levels indicated in animal experiments by an increase in hypocretin‐1 could be due to abnormal glycolysis, we determined the glycolysis rate. In the glycolysis rate test, exogenous hypocretin‐1 decreased both the basal glycolysis rate and the compensatory glycolysis rate after adding Rotenone/Antimycin A (Rot/AA) of astrocytes, and significantly decreased the extracellular acidification rate (ECAR) (Figure 4e–g), which was consistent with the reduction in lactate levels, indicating hypocretin‐1 could significantly reduce lactate production in astrocytes. Simultaneously, mRNA expression of GLUT1, MCT1, MCT4 and LDHA in astrocytes was significantly reduced by hypocretin‐1, while the ratio of LDHA to LDHB significantly decreased, which was reversed in the SB‐334867 group (Figure 4d). Notably, hypocretin‐1 also significantly reduced the mRNA expression of HIF‐1α in astrocytes (Figure 4d), immunofluorescence staining showed similar results, which could be improved by pre‐administration of SB‐334867 (Figure 4h,i). We further investigated the role of HIF‐1α in HCRT‐1‐induced reduction of lactate (Figure 4j). The addition of HIF‐1α agonist to astrocytes revealed that it was able to reverse the HCRT‐1‐induced disruption of the expression of glycolytic pathway‐related factors (GLUT1, MCT1, MCT4, LDHA, and LDHB) (Figure 4k). These findings provide more directly evidence that HCRT‐1 reduces the expression of glycolysis‐related factors and lactate release in astrocytes may through regulation of HIF‐1α.

**Figure 5:**
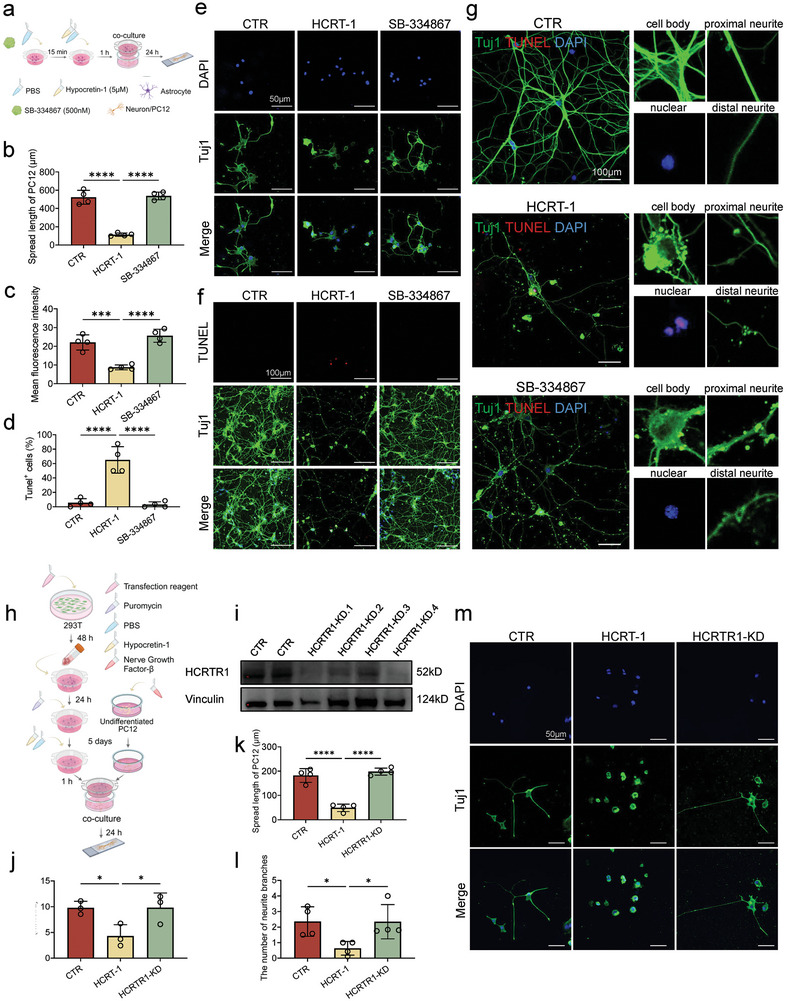
Hypocretin‐1 stimulated astrocytes caused neuronal damage. a) Schematic diagram showing the co‐culture of astrocyte and neuron or PC12. SB‐334867, a HCRTR1 antagonist. b) The effect of hypocretin‐1 in neuron speed length in PC12 cell lines. c,d) Quantification of mean fluorescence intensity of Tuj1 (beta III tubulin) and Tunel^+^ (TdT‐mediated dUTP Nick‐End Labeling) cells in the primary neuron. e) Representative microscopic fields of PC12 co‐cultured with astrocytes in different groups. f,g) Representative microscopic fields of neuronal apoptosis and neurite branches in primary hippocampus neurons. h) Schematic diagram showing the knockdown of HCRTR1 in astrocytes and co‐culture of astrocyte and PC12. i) Validation of successful knockdown of HCRTR1 in astrocytes. j) The lactate in astrocyte medium in different groups. k,l) Quantification of speed length and the number of neurite branches in PC12 cell lines. m) Representative microscopic fields of PC12 co‐cultured with astrocytes in different groups. Dots in panels represent individual samples. Data were shown as mean ± SD. One‐way ANOVA followed by Dunnett's post hoc test. *p < 0.05, **p < 0.01, ***p < 0.001, ****p < 0.0001; ns, no significant difference, *p *≥ 0.05.

Lactate can increase neuronal excitement.^[^ ^27^ ^]^ To further confirm the effect of hypocretin‐1‐induced reduction of lactate level in astrocytes on neurons, we co‐cultured astrocytes with PC12 cell lines and primary neurons, respectively (**Figure** **5**a). It was found that PC12 co‐cultured with HCRT‐1‐stimulated astrocytes had shorter axons, and this was reversed in the SB‐334867 group (Figure 5b,e). This provided evidence that HCTR‐1 induced astrocytes could damage neuronal axons. Additionally, an increased proportion of apoptotic death and damage to neuron body and distal neurite were found in hippocampal neurons co‐cultured with astrocytes given HCRT‐1 (Figure 5c,d,f,g). In contrast, pre‐administration of SB‐334867 was sufficient to reverse, reducing the proportion of neuronal apoptosis and maintaining their normal morphology successfully (Figure 5c,d,f,g).

Additionally, lentivirus was used to knock down HCRTR1 in astrocytes (Figure 5h), and the knockdown (KD) effect of different sequences was verified by WB, and the best knockdown sequence HCRTR1‐KD.4 was determined (Figure 5i). Consistent with the prior administration of antagonists, co‐culture of astrocytes with PC12, the HCRTR1‐KD group was able to reverse reduction release of lactate (Figure 5i) and the axon shortening and neurite branches reduction induced by HCRT‐1 (Figure 5j–l). These results provide further support that HCRT‐1‐induced reduced lactate release from astrocytes leads to hippocampal neuron damage, which may further lead to impaired cognitive function in individuals with depression.

### 2.6 Astrocytic HCRTR1 Knockdown Ameliorates Anxiety‐Depressive‐Like Behavior and Cognitive Impairment

**Figure 6:**
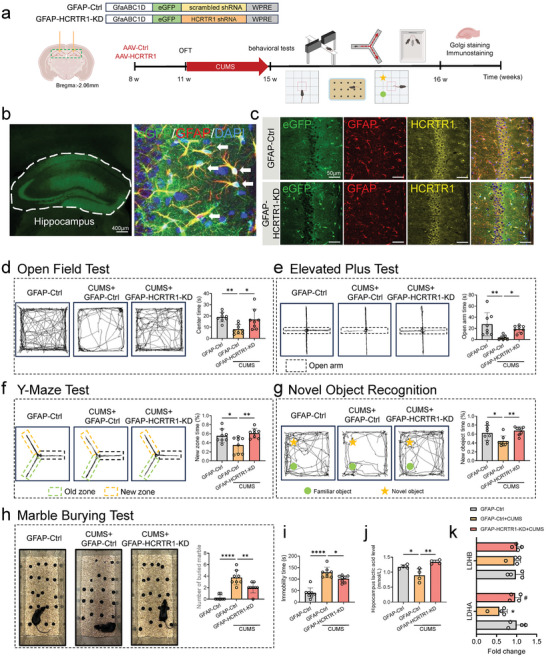
Knockdown of HCRTR1 in hippocampal astrocytes reversed CUMS‐induced anxiety and depressive‐behaviors and cognitive repairment. a) The procedure of animal model establishment. CTR, GFAP‐Ctrl; CUMS, GFAP‐Ctrl + CUMS; GFAP‐HCRTR1‐KD, GFAP‐HCRTR1‐KD + CUMS. b) Illustration of viral injections. Solid arrows, eGFP and GFAP. c) Validation knockdown of HCRTR1 in hippocampal astrocytes. d) The exploration time in center area in open field test in different groups. e) The exploration time in open arm in the elevated plus test in different groups. f) The proportion of time exploring in new zone in Y‐maze in different groups. g) The proportion of time exploring in novel object in novel object recognition test in different groups. h) The number of buried marbles in marble burying test different groups. i) The immobility time in tail suspension test in different groups. j) The lactate level in hippocampus in different groups. k) The mRNA expression of LDHA and LDHB in hippocampus different groups. Dots in panels represent individual samples. Data were shown as mean ± SD. One‐way ANOVA followed by Dunnett's post hoc test. *p < 0.05, **p < 0.01, ***p < 0.001, ****p < 0.0001; ns, no significant difference, *p *≥ 0.05.

To determine whether hippocampal astrocytic HCRTR1 is involved in the regulation of depressive‐like behavior and cognitive impairment, we achieved astrocytic HCRTR1 knockdown by bilateral injection of adeno‐associated virus (AAV) vectors expressing a short hairpin RNA (shRNA), specifically targeted HCRTR1 transcripts (GFAP‐HCRTR1 shRNA) into the hippocampus (**Figure** **6**a,b). Immunofluorescence results showed reduced HCRTR1 expression in the hippocampal astrocytes of GFAP‐HCRTR1‐shRNA (termed GFAP‐HCRTR1‐KD) mice compared to control (GFAP‐Ctrl) mice (Figure 6c). Before the CUMS procedure, there was no difference in center time and total distance in OFT between GFAP‐Ctrl and GFAP‐HCRTR1‐KD groups (Figure S3, Supporting Information). Behaviorally, compared to GFAP‐Ctrl + CUMS group, GFAP‐HCRTR1‐KD group exhibited longer central area and open arm exploration time in OFT and EPM, respectively (Figure 6d,e). Meanwhile, GFAP‐HCRTR1‐KD group exhibited fewer buried marbles in the marble burying test and shorter immobility time in the TST (Figure 6h,i). Notably, GFAP‐HCRTR1‐KD also reversed the CUMS‐induced reduction in exploration of new arms and objects in the Y‐maze and NOR (Figure 6f,g). These results all suggest that HCRTR1 in the hippocampus astrocytes plays an important role in modulating depressive‐like behavior and cognitive impairment.

**Figure 7:**
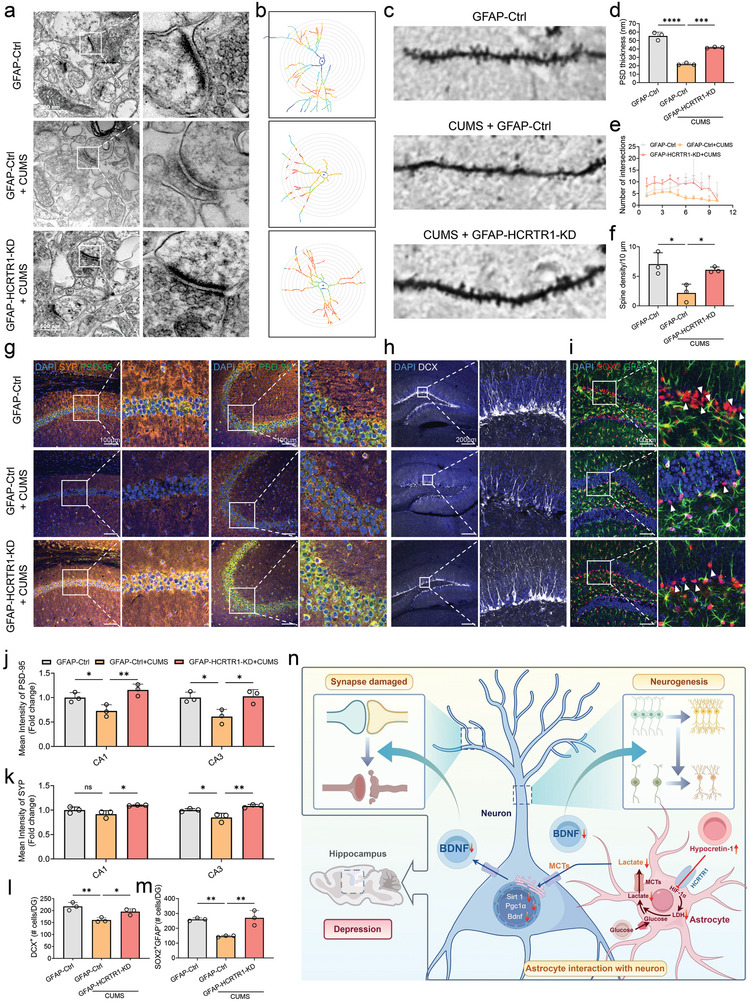
Knockdown of HCRTR1 in hippocampal astrocytes reversed CUMS‐induced hippocampal synaptic plasticity damage. a) Representative transmission electron microscope images showing the synaptic structure and postsynaptic densities in hippocampus. scale bar, 500 nm. b,c) Typical image of Golgi staining. d) Quantification of postsynaptic density thickness in different groups. e,f) Quantification of the number of intersections and spine density in different groups. g–i) Representative microscopic of PSD‐95 and SYP, DCX positive cells and GFAP and SOX2 positive cells within the hippocampus in different groups, respectively. scale bar, 100 µm. j,k) Quantification of mean fluorescence intensity SYP and PSD‐95, respectively. l) Quantification of DCX positive cells in the DG. scale bar, 200 µm. m) Quantification of SOX2^+^/GFAP^−^ cells in the DG. n) Hypocretin‐1 in depression may negatively regulate the HIF‐1α pathway through HCRTR1, disrupting the glycolytic pathway and resulting in the reduction lactate release from astrocytes, thereby disrupting synaptic plasticity and neurogenesis of hippocampal neurons, which may be lead to cognitive impairment in depressed individuals. Dots in panels represent individual samples. Data were shown as mean ± SD. One‐way ANOVA followed by Dunnett's post hoc test. *p < 0.05, **p < 0.01, ***p < 0.001, ****p < 0.0001; ns, no significant difference, *p *≥ 0.05.

Furthermore, CUMS‐induced dysregulation of hippocampal lactate and LDHs levels were also restored in GFAP‐HCRTR1‐KD (Figure 6j,k). Transmission Electron Microscopic (TEM) results also showed that CUMS‐induced damage to the postsynaptic dense area in the hippocampus was also ameliorated in the GFAP‐HCRTR1‐KD group (**Figure** **7**a,d). Additionally, the expression of key synaptic markers (SYP and PSD‐95) was significantly increased in the hippocampus by GFAP‐HCRTR1‐KD, compared to the CUMS group (Figure 7g,j,k; Figure S4, Supporting Information). Dendritic spines on hippocampal neurons are the main structural basis of excitatory synapses, and changes in dendritic spine density are critical for postsynaptic plasticity and contribute to the morphological basis of learning and memory functions.^[^ ^57^ ^]^ Golgi staining was performed to further investigate the effect of GFAP‐HCRTR1‐KD on neuronal dendritic spines. The results showed that GFAP‐HCRTR1‐KD reversed the CUMS‐induced fewer dendritic crossings, shorter total dendritic length, and reduced dendritic spine density (Figure 7b,c,e,f). The restoration of neuroplasticity was further confirmed by the significant increase in the number of DCX^+^ and SOX2^+^GFAP^−^ cells observed in GFAP‐HCRTR1‐KD group (Figure 7h,i,l,m). Meanwhile, CUMS‐induced decreased HIF‐1α mRNA expression in hippocampus was also significantly increased in GFAP‐HCRTR1‐KD (Figure S5b, Supporting Information), as well as PGC‐1α‐Sirt1‐BDNF pathway‐related factors (Figure S5a, Supporting Information), which provides further evidence that the hypocretin system regulates lactate release through negative regulation of HIF‐1α. Collectively, these findings also provide compelling evidence for the pivotal role of HCRTR1 in hippocampal astrocytes in modulating lactate production, synaptic plasticity, and thereby mitigating depressive‐like behaviors and cognitive impairment.

### 2.7 Lactate Reversed Hypocretin‐1 Induced Cognitive Impairment

Lactate homeostasis plays a vital role in depression,^[^ ^58^ ^]^ we further investigate whether lactate could ameliorate hypcretin‐1 induced cognitive impairment in depression. The results showed that icv.HCRT‐1 showed a decreased ratio of new zone and new object exploration time in the Y‐maze and NOR, respectively, compared with the CTR group, while icv.HCRT‐1+lactate significantly reversed (Figure S6, Supporting Information), suggesting that lactate could significantly ameliorate the cognitive impairment induced by hypcretin‐1.

## 3 Discussion

Glycolysis and lactate shuttling play a pivotal role in the regulation of brain physiology. Multiple lines of evidence suggest that peripheral administration of exogenous L‐lactate exhibits antidepressant effects in both human patients and animal models.^[^ ^59^ ^]^ Astrocytes are responsible for maintaining lactate homeostasis within the central nervous system,^[^ ^58^ ^]^ yet the precise mechanism by which astrocytes regulate lactate in depression remains elusive. In this study, we illustrate that elevated levels of hypocretin‐1 suppress lactate production and release from astrocytes, consequently impairing neurogenesis and synaptic plasticity. These alterations ultimately contribute to depressive‐like phenotypes such as depressed mood and cognitive deficits. Importantly, we reveal that this effect is mediated through the HCRTR1‐HIF‐1α pathway. Furthermore, our findings indicate that lactate may modulate neurogenesis and synaptic plasticity by influencing BDNF transcription (Figure 7n).

In our prior study, the intensity of immunostaining in hypothalamic hypocretin‐1 neurons was significantly higher in MDD patients than in controls.^[^ ^13^ ^]^ High levels of hypocretin‐1 may be involved in cognitive impairment in depression. Notably, the elevated hypocretin‐1 level of cerebrospinal fluid in patients with Alzheimer's disease are associated with rapid cognitive decline.^[^ ^16^ ^]^ We demonstrated that hypocretin‐1 levels were significantly elevated in both plasma and hypothalamus in a CUMS model with cognitive impairment, along with mRNA expression of HCRTR1 in the hippocampus. However, HCRTR1 antagonists and GFAP‐HCRTR1‐KD both reversed cognitive impairment in CUMS mice. Similarly, icv.HCRT‐1 rats also exhibited depressive‐like behavior and showed cognitive impairment in the Y‐maze, which consistent with previous studies showing that rats injected with hypocretin‐1 showed impaired spatial learning and memory function, accompanied by altered synaptic plasticity and reduced long‐term potentiation.^[^ ^18^ ^]^ These suggest that changes in hypocretin‐1/HCRTR1 may be linked to cognitive impairment in depression.

Even in the absence of hypocretin‐1 treatment, hypocretin receptor‐containing neurons exhibited lower levels of LDHA compared to cells isolated from HCRTR1 knockout mice, which was further potentiated by hypocretin‐1 treatment, resulting in a more pronounced reduction in LDHA expression, particularly in HIF‐1α‐KD cells.^[^ ^60^ ^]^ We demonstrated that hypocretin‐1 suppressed HIF‐1α expression and subsequent LDHA expression in astrocytes, and this effect could be reversed by HIF‐1α agonist. Increased HIF‐1α activity led to a significant enhancement in glucose utilization and glycolytic activity. In astrocytes, reduction of HIF‐1α levels suppressed the expression of downstream target genes implicated in glucose transport and glycolysis.^[^ ^61^ ^]^

Studies conducted in human and animal models have demonstrated that depression and chronic stress are associated with altered synaptic plasticity, characterized by a reduction in the density of postsynaptic dendritic spines and decreased expression of genes related to synapses in the prefrontal cortex and hippocampus.^[^ ^33^, ^62^, ^63^, ^64^ ^]^ Interestingly, lactate has been found to enhance the expression of genes involved in synaptic plasticity, potentially ameliorating depressive symptoms and facilitating memory formation by maintaining normal synaptic function.^[^ ^45^, ^65^, ^66^ ^]^ Furthermore, peripheral administration of lactate has shown antidepressant effects as well as cognitive improvement.^[^ ^59^, ^67^ ^]^ Consistent with these findings, our study revealed downregulation of lactate levels in the hippocampus under CUMS; however, this effect was reversed by treatment with HCRTR1 antagonists or GFAP‐HCRTR1‐KD intervention. There is growing evidence suggesting impaired glucose metabolism within the hippocampus of both depressed patients and animal models.^[^ ^23^, ^68^ ^]^ Our findings suggest that disrupted glycolytic pathways (Gluts, MCTs, LDHs) in CUMS are able to be restored by HCRTR1 antagonists and GFAP‐HCRTR1‐KD. Furthermore, the direct injection of hypocretin‐1 into the hippocampus also caused a decrease in the level of lactate in the hippocampus of rats. Similarly, exposure to CUMS and icv.HCRT‐1 led to decreased glucose uptake and lactate production, due to changes in the glycolytic pathway (Gluts, MCTs, LDHs), as more directly confirmed by microPET. These results suggest that hypocretin‐1 may damage the glycolytic pathway, triggering a decrease in lactate production, leading to neuroplasticity disorder and cognitive impairment. Interfering with lactate transport in hippocampal astrocytes, or inhibiting lactate release, would disrupt long‐term memory formation.^[^ ^45^, ^65^ ^]^ Therefore, Reduced lactate production and release mediated by elevated hypocretin‐1 levels may contribute to depression and cognitive impairment.

Notably, we further investigated the mechanism by which lactate influences cognition. Lactate has the potential to enhance levels of BDNF, a protein highly expressed in the brain that regulates neuronal plasticity, thereby promoting learning and memory as well as neuroprotection.^[^ ^49^, ^69^, ^70^, ^71^ ^]^ Consistently, our results demonstrated that HCRTR1 antagonists ameliorated cognitive impairment induced by CUMS while reversing the decline in hippocampal lactate levels and BDNF expression, which exhibited a positive correlation. The generation of protective BDNF necessitates identification of appropriate targets; here we considered the role of Sirt1, whose activation plays a crucial role in neuroprotection and improved cognition.^[^ ^72^, ^73^ ^]^ Additionally, plasma BDNF and Sirt1 levels are positively correlated in patients with depression.^[^ ^74^ ^]^ Lactate regulates the redox state of neurons through alteration the NAD/NADH ratio,^[^ ^75^ ^]^ leading to activation of Sirt1 whose activity is dependent on NAD levels. Previous studies have indicated that PGC‐1αalso acts as an important mediator for hippocampal BDNF induction.^[^ ^76^ ^]^ Furthermore, lactate can facilitate PGC‐1α protein production in a Sirt1‐dependent manner.^[^ ^50^ ^]^ In our study, in line with lactate depletion, Sirt1, PGC‐1α, and BDNF levels were significantly reduced in the CUMS group exhibiting cognitive impairment but were upregulated following treatment with HCRTR1 antagonists. This suggests that lactate may induce an increase in BDNF expression by upregulating PGC‐1α and Sirt1 levels, thereby enhancing cognition.

Previous studies have reported that hypocretin‐1 is associated with psychiatric disorders, including bipolar disorder,^[^ ^14^, ^77^ ^]^ schizophrenia,^[^ ^78^, ^79^ ^]^ depression.^[^ ^13^, ^14^, ^15^, ^80^ ^]^ Additionally, a broader role for hypocretin‐1, including in addiction^[^ ^81^, ^82^ ^]^ and sleep disorders,^[^ ^83^ ^]^ which have still provided us with valuable insights. However, we have found that the mechanisms by which hypocretin acts can differ across diverse disease states and even between distinct brain regions. In general, the current work represents a relatively systematic investigation into the mechanistic underpinnings of hypocretin's involvement in a mouse model of depression. By establishing a variety of in vivo models, including the CUMS model, and directly intervening in hypocretin‐1 or establishing models of HCRTR1 knockdown in the hippocampus, we further explored the specific mechanism of hypocretin‐1 involvement in depression, Our data suggest that hypocretin‐1 regulates the glycolytic pathway, and that HCRTR1 affects the reduction of lactate release from hippocampal astrocytes and impairs synaptic plasticity and neurogenesis, thus playing an important role in depressive cognitive dysfunction. Building on previous researches, we further explored the specific mechanism of hypocretin‐1 contribution to depression, that established a variety of in vivo models, including the CUMS‐model, icv. hypocretin‐1 model, and hippocampal GFAP‐HCRTR1‐KD model, which demonstrated HCRTR1 affects the reduction of lactate release from hippocampal astrocytes and impairs synaptic plasticity and neurogenesis, thus playing an important role in cognitive dysfunction in depression. In addition, though HCRTR1 and its inhibitor SB‐334867 have indeed been preliminarily explored in the treatment of psychiatric disorders, including the amelioration of depressive‐like behaviors induced by various models,^[^ ^84^, ^85^, ^86^ ^]^ the potential therapeutic effects of cocaine‐addiction‐related phenotypes,^[^ ^81^, ^82^ ^]^ we not only investigated the role of SB‐334867 in ameliorating anxiety and depressive‐behaviors, but also in improving cognitive function. Following the association between hypocretin‐1 and HIF‐1α^60^, simply providing cellular‐level validation, we further found that HIF‐1α may be negatively regulated by hypocretin‐1, disrupting the glycolytic pathway and becoming a potential target for the treatment of cognitive dysfunction in depression. However, we have not directly investigated changes in lactate levels and the association between lactate levels and cognitive performance in patients with depression, which is one of the limitations of this study.

In summary, we investigated the role of hypocretin system in cognitive impairment in depressive‐like mice and demonstrated that hypocretin‐1 plays an important role in cognitive impairment in depression by regulating the glycolytic pathway and affecting lactate production through HCRTR1.

## 4 Experimental Section

### Animals

The local animal care committees approved all procedures in accordance with relevant regulations and laws. I) 8‐week‐old male C57BL/6J mice were randomly divided into the control (vehicle) group, chronic unpredictable mild stress model group (CUMS + vehicle), SB‐334867 group (CUMS + SB‐334867 (#HY‐10895, MCE), 5 mg kg^−1^). SB‐334867 group were CUMS mice treated with SB‐334867 during the last four weeks, by intraperitoneal injection, respectively. *n* = 8 in each group. II) 8‐week‐old male C57BL/6J mice were randomly divided into the control (vehicle) group and SB‐334867 group (control + SB‐334867, 5 mg kg^−1^, treatment for 4 weeks). III) 8‐week‐old male SD rats were randomly divided into control, CUMS and icv.HCRT‐1 (3 nmol, #HY‐106224, MCE) groups. *n* = 6 in each group. IV) 8‐week‐old male C57BL/6J mice were randomly divided into the control group (GFAP‐Ctrl), CUMS group (CUMS + GFAP‐Ctrl), GFAP‐HCRTR1‐KD group (CUMS + GFAP‐HCRTR1‐KD). *n* = 8 in each group. V) 8‐week‐old male C57BL/6J mice were randomly divided into the control (icv. vehicle), icv.HCRT‐1 (0.3 nmol, #HY‐106224, MCE) and icv.HCRT‐1+lactate (#HY‐B2227, MCE, 10 mg kg^−1^, treating for continuous 3 days) groups. The day after the end of the behavior, the rodents are sacrificed. Part of the mice and rats were anesthetized with isoflurane (2.5% and 4%, respectively) for cardiac blood collection and sacrificed. The hippocampal tissues were separated from the brain as well. Plasma was obtained after centrifugation at 3000 rpm for 10 min. Plasma and tissues were stored at ‐80 °C until use. The remaining rodents were anesthetized by intraperitoneal injection of pentobarbital (60 mg kg^−1^) and then perfused PBS through the heart, then infused with 4% PFA. The brain was separated and fixed with 4% PFA for more than 24 h. After brain dehydration and embedding, 4 µm paraffin sections or 40 µm frozen section were made.

### Stereotactic Surgery and Hypocretin‐1 or Virus Injection

Adult SD male rats were kept anesthetized with 2.5% isoflurane or adult C57BL/6J male mice were kept anesthetized with 1.5% isoflurane and head‐fixed in a stereotaxic device (RWD Life Science. Inc). A specific volume of hypocretin‐1 was injected for rat (coordinates from bregma: AP, −1.1 mm; ML, ±1.5 mm; DV, −4.5 mm) and mouse (coordinates from bregma: AP, −0.46 mm; ML, ± 1.0 mm; DV, −2.0 mm), respectively. For virus injection to mice, 300 nL of the virus was injected bilaterally into the hippocampus (coordinates from bregma: AP, −2.0 mm; ML, ±1.5 mm; DV, −1.5 mm). Both hypocretin‐1 and virus were injected at a rate of 10 nL sec^−1^, capillary will be slowly retracted 10 min after injection.

The recombinant AAV vectors were constructed and packed by OBiO Technology (Shanghai, China). Vectors used in the present study were as followed: GFAP‐HCRTR1‐KD (AAV2/5‐GfaABC1D‐ HCRTR1‐shRNA‐eGFP, titre: 5.81 × 1012v.g. mL^−1^, 300nL bilateral into hippocampus) and GFAP‐Ctrl (AAV2/5‐GfaABC1D‐scrambled‐shRNA‐eGFP, titre: 1.0 × 1012v.g. mL^−1^, 300nL bilateral into hippocampus). The shRNA target sequences of HCRTR1: GCATCAAGAGCACTGTTAAGA.

### CUMS Model

The procedure for CUMS follows similar to that in the previous study.^[^ ^12^, ^13^, ^87^ ^]^ Simply, rodents were randomly exposed to the following stimuli for 4 weeks: 1) deprivation of food and water for 24 h; 2) restraint stress for 6 h; 3) odor stimulation for 24 h; 4) wet pad for 24 h; 5) clip tail 60 s; 6) cold water swimming (4 °C) for 5 min; 7) cage tilted (45°) for 6 h; 8) no padding for 24 h; 9) shake for 10 min; 10) strobe for 12 h; 11) light/dark cycle reversed for 24 h; 12) white noise for 12 h. The control group was not exposed to the CUMS procedure, without being disturbed except for the necessary procedures.

### Behavioral Tests

Behavioral tests were performed to evaluate the behavioral impairment in different groups. The detailed procedures are shown in Supporting Information.

### Micro‐PET

^18^F‐FDG microscopic PET /CT imaging was acquired using high‐resolution microscopic PET /CT (SuperArgus, Spain). After deprivation of food for 8 h, the rats were given ≈18.5 MBq (500 µCi) of ^18^F‐FDG intravenously, anesthetized with 5% isoflurane after 20 min, and maintained under anesthesia with 3% isoflurane. PET images for 10 min were collected and CT for 2 min scan was performed to collect anatomical information.

The PET and CT images were registered with PMOD software (version 3.902, PMOD Technologies Ltd.). The volume of interest (VOI) of the hippocampus and pons was mapped using the rat brain template encapsulated by PMOD software (W. Schiffer). The pons was used as the reference area. The standard absorption value ratio (SUVR) was used for semi‐quantitative analysis. SUVR = SUV (hippocampus)/ SUV (pons).

### Enzyme‐Linked Immunosorbent Assay (ELISA) Assay

According to the manufacturer's instructions, plasma and hypothalamic hypocretin‐1 level, plasma and hippocampal BDNF were measured using ELISA kits (hypocretin‐1, CUSABIO, CSB‐E08861m; BDNF, AiFang biological, AF2204‐A), respectively. which were determined by measuring absorbance at 450 nm using a standard curve.

### Nissl Staining

Nissl staining was used to determine neuronal damage in the hippocampus. Briefly, according to the working instructions, toluidine blue staining solution was used to stain dewaxed hippocampal sections for 10 min. Sections were imaged under a digital trinocular camera microscope (BX53, Olympus) after dehydration, hyalinization, and mounting.

### Golgi‐Cox Staining and Analysis

Golgi‐Cox staining was performed using the FD Rapid GolgiStain kit. Mice were executed by decapitation 24 h after behavioral tests, and brain tissue was rapidly collected in MilliQ. Their anterior parts were dissected and immersed in freshly prepared Golgi‐Cox buffer for 2 weeks, and the brain tissue was cryoprotected in cryoprotectant buffer (4 °C) for 1 week before being frozen in liquid nitrogen and made into 100 µm sections. Sections were mounted on gelatin‐coated slides, dried at room temperature for 72 h, and stained according to the manufacturer's instructions.

Tissue information was collected using a PANNORAMIC panoramic section scanner, and the target area of brain tissue was identified for imaging using SlideViewer2 scanning software, ensuring that the background light and magnification of each photo were consistent, and each central neuron cytosolic structure was mapped using ImageJ analysis software, with 10 concentric circles at 10 µm distance centered on the cytosol. The length was measured and the number of dendritic spines in the length was counted, and take the number of dendritic spines per 10 µm was taken as its density = number of dendritic spines/length of dendrites×10.

### Immunofluorescent Staining of Mice Hippocampus

Paraffin sections were dewaxed (xylene 10 min, twice; 100%, 100%, 96%, 90%, 80%, 70%, 60%, 50% ethanol, 5 min, respectively), subjected to antigen repair, naturally cooled, and blocked with 3% BSA for 1 h at room temperature. Frozen sections were washed in PBS and blocked with 3% BSA for 1 h at room temperature. Sections were incubated with different primary antibodies (postsynaptic density protein‐95, PSD‐95, #20665‐1‐AP, Proteintech; synaptophysin, SYP, #67864‐1‐Ig, Proteintech; SOX2, # ab93689, Abcam; glial fibrillary acidic protein, GFAP, # ab7260, Abcam; doublecortin, DCX, #ab18723, Abcam; HCRTR1, #NBP2‐95106, Novus) overnight at 4 °C. Next, were incubated with the corresponding secondary antibodies at room temperature and protected from light for 1 h. The sections were blocked with an anti‐fluorescence quenching blocker.

### TEM Analysis

A piece of hippocampal tissue from the CA3 region was immersed in 2.5% glutaraldehyde and fixed overnight. The tissues were then fixed in 1% osmium tetroxide for 2 h at room temperature and washed 3 times in PBS, afterward, were sequentially dehydrated into graded ethanol (50%, 70%, 80%, 90%, 95%, 100% I, 100% II, respectively, for 15 min) and 100% acetone (twice, for 30 min), embedded in Epon and cured overnight at 37 °C. Ultrathin sections were stained with lead citrate and observed with a transmission electron microscope (Tecnai G2 spirit 120v) to collect ultrastructural images of synapses in the hippocampus.

### Primary Neuron and Astrocyte Cultures

For primary astrocyte culture, hippocampal tissue was isolated from 0–1 day old C57BL/6J mice under aseptic conditions and digested with 0.25% trypsin for 15 min at 37 °C. The isolated cells were inoculated with the media (DMEM/F‐12, #11 320 033 Gibco, supplemented with 15% fetal bovine serum, #F801‐500, BDBio, China) and 1% penicillin/streptomycin (#15 140 122, Gibco) into poly‐D‐lysine (PDL, #P6407, Sigma‐Aldrich)‐pretreated T75, and the medium was changed every other day. 10–12 days later, flasks were shaken to purify astrocytes (37 °C, 200 rpm, 12 h).

For primary neuronal cultures, hippocampal tissue was isolated from E18 C57BL/6J mice and digested with 0.25% trypsin for 15 min at 37 °C. The isolated cells were resuspended in neurobasal media (#21 103 049, Gibco) supplemented with 2.5% inactivated fetal bovine serum, 2% B27 (#17 504 044, Gibco), and 1% Glutamax (#35 050 061, Gibco) and inoculated into PDL‐pretreated dishes for 4 h and then replaced with the maintenance medium (neurobasal media supplemented with 2% B27 and 1% Glutamax) was changed every 3–4 days at half intervals.

For co‐culture, primary astrocytes were inoculated on a cell culture insert (Costar, #3460) and first exposed to the following stimuli for 1 h: (I) equal amounts of PBS; (II) Hypocretin‐1 (5 µm, #HY‐106224, MCE); (III) Hypocretin‐1 (5 µm) plus SB‐334867 (HCRTR1 antagonist, 500 nm, #HY‐10895, MCE, 15 min before). Subsequently, moved to co‐culture with primary neurons for 24 h.

### Glycolysis Analysis

The ECAR was measured using an XFe24 analyzer (Agilent) and the seahorse XF glycolysis rate kit (Agilent, #103344‐100). Astrocytes (4 × 10^4^ /well) were inoculated in 24‐well Seahorse XF microtiter plates with medium and incubated overnight at 37 °C and 5% CO_2_. After drug treatment (PBS, hypocretin‐1 and hypocretin‐1 plus SB334867, respectively), the medium was replaced with analytical medium (#103575‐100, Seahorse XF DMEM Medium) supplemented with 1 mm pyruvate (#103578‐100), 2 mm glutamine (#103579‐100) and 10 mm glucose (#103577‐100). Cells were incubated in a non‐CO_2_ incubator at 37 °C for 1 h. Rot/AA (0.5 µm) and 2‐deoxy‐D‐glucose (50 mm) were added to the XFe24 probe plate and assayed.

### shRNA Experiment in Astrocyte

To knockdown HCRTR1 in astrocytes, lentiviruses were used to infect primary astrocytes with 70% confluence. Puromycin (0.2 ug mL^−1^) was added to the complete medium 24 h after lentiviruses infection and the medium was changed every other day for 5 days before subsequent experiments. Targeted sequences for HCRTR1 shRNA: GCATCAAGAGCACTGTTAAGA.

### PC12 Cells Cultures

PC12 cells were derived from rat adrenal pheochromocytoma (iCell‐r025, iCell Bioscience Inc, Shanghai, China), cultured in 1640 medium (#11 875 093, Gibco), supplemented with 5% fetal bovine serum, 10% inactivated horse serum and 1% penicillin/streptomycin, cryopreserved in CELLSAVING (#C40050, NCM biotech) and stored at −80 °C. Cells were spread in PDL‐pretreated wells and after 1day primary culture was replaced with differentiation medium (containing 100 ng mL^−1^ nerve growth factor, #HY‐P700162AF, MCE) and differentiated for 5 days. In co‐culture experiments, astrocytes were inoculated on cell culture inserts (Costar, #3460). Before being transferred to differentiated PC12 cells for co‐culture for 24 h, astrocytes were exposed for 1 h to the following media: (I) equal amounts of PBS; (II) Hypocretin‐1 (5 µm); (III) Hypocretin‐1 (5 µm) plus SB‐334867 (HCRTR1 antagonist, 500 nm, 15 min before).

### Immunofluorescent Staining

Cells inoculated on the round coverslip were collected and fixed in 4% PFA for 15 min and incubated with 0.1% Triton X‐100 for 15 min at room temperature, subsequently, were closed with 2% BSA at 37 °C for 30 min. Incubation with different primary antibodies overnight at 4 °C (GFAP, #ab7260, Abcam; beta III tubulin, Tuj1, #66375‐1‐Ig, Proteintech), followed by incubation with the corresponding secondary antibodies at room temperature and protected from light for 1 h and blocked with an anti‐fluorescence quenching blocking agent (#S2110, Solarbio). In addition, neurons were marked with TdT‐mediated dUTP Nick‐End Labeling (TUNEL, #40307ES60, YEASEN) according to the manufacturer's instructions to distinguish apoptotic neurons. Specific fluorescent signals were tested using a confocal microscope (FV3000).

### Extracellular Lactate Measurement

Lactate level was measured according to the manufacturer's instructions (#A019‐2‐1, Nanjing Jiancheng Bioengineering Institute). Briefly, hippocampal tissue was taken and homogenized mechanically under ice bath conditions after addition of PBS at 1:9 (v/v), centrifuged at 12,000 rpm for 10 min and the supernatant collected. After administration, the astrocyte medium was collected and the supernatant was collected after centrifugation at 12,000 rpm for 10 min.

### Quantitative Real‐Time PCR (qRT‐PCR) Analysis

Briefly, the total RNA of hippocampal tissue and astrocytes was extracted by Fast Pure Total RNA Isolation Kit (#RC112‐01, Vazyme) and reverse transcribed to cDNA (#R333‐01, Vazyme). qRT‐PCR was performed using PerfectStart Green qPCR SuperMix (#AQ601, TransGen Biotech). The 2^−ΔΔCt^ method was utilized to determine the target gene mRNA levels, which were normalized to those of β‐actin. Primer sequences are shown in Supporting Information.

### Western Blot

Hippocampal tissue was fully lysed with RIPA lysate containing phosphatase and protease inhibitors on a freeze grinder, then centrifuged at 12 000 rpm at 4 °C for 15 min, and the supernatant was transferred to a clean centrifuge tube. Protein samples were isolated using 4–20% SDS‐PAGE (#M00657, SurePAGE, GenScript) and transferred to PVDF membranes using eBlot L1 (#L00686C, Genscript), incubated in 5%BSA at room temperature for 1 h before incubation of primary antibody (4 °C, overnight; Vinculin, #13 901, cell signaling technology, 1:1000; HCRTR1, #18 370, Proteintech). After washing and incubation at room temperature for 1 h with the corresponding secondary antibodies, bands were visualized using enhanced chemiluminescence reagents (#SW134, Seven/Abcells) and quantified by image J. β‐actin was used as the loading control.

### Statistical Analysis

Statistical analyses were performed using GraphPad Prism, version 9.3.1 (GraphPad Software). Data were presented as mean ± SD for normally distributed variables. Differences among experimental groups were determined by one‐way ANOVA followed by Dunnett's test for post‐hoc comparisons. Non‐normally distributed data were presented as mean ± SEM and were compared between the experimental groups by Kruskal‐Wallis test followed by Dunnett's test for post‐hoc comparisons. *p* < 0.05 was considered statistically significant.

### Ethics Approval

All animal studies were performed in accordance with guidelines approved by the Animal Experimentation Ethics Committee of the First Affiliated Hospital, Zhejiang University School of Medicine.

## Conflict of Interest

The authors declare no conflict of interest.

## Author Contributions

B.C. and K.J. contributed equally to this work. J.L. performed conceptualization, funding acquisition, supervision, and wrote, reviewed, and edited the draft. K.J. and B.C. performed conceptualization, methodology, visualization, wrote the original draft, wrote, reviewed, and edited the draft. J.D. and S.C. performed methodology. Z.C. performed funding acquisition, supervision, and wrote, reviewed, and edited the draft. S.H. performed supervision and wrote, reviewed, and edited the draft. L.K. performed wrote, reviewed, and edited the draft.

## Supporting information

Supporting Information

## Data Availability

The data that support the findings of this study are available from the corresponding author upon reasonable request.
