# Supplementary material for: Hypocretin‐1/Hypocretin Receptor 1 Regulates Neuroplasticity and Cognitive Function through Hippocampal Lactate Homeostasis in Depressed Model
Source: Adv Sci (Weinh). 2024 Aug 9;11(38):2405354. doi: 10.1002/advs.202405354 (PMC11481194; doi:10.1002/advs.202405354)
Supplement: Supplementary file 1 — Supporting Information [file ADVS-11-2405354-s001.docx]

**Behavior Test**

**Open field test (OFT)**

The OFT is a measure of anxiety-like behavior. Each mouse was placed in a white arena (50×50×50 cm), with a center region of 12.5×12.5 cm, and the detail was monitored for 5 min by an overhead camera and tracking software, while the rats were placed in a black open box (100×100×100 cm) with a central area of 25×25 cm. The duration of mice and rats active in the central region is a key focus.

**Elevated plus maze (EPM)**

Elevated plus maze, as a measure of anxiety-like behavior, is placed 50 cm above the ground, and consists of 4 arms (2 open arms and 2 closed arms) of 5 cm in width, 35 cm in length and the closed arms height of 15 cm. The elevated plus maze for rats consists of 4 arms of 10 cm in width, 50 cm in length and the closed arms height of 40 cm. During the test, the mice were placed in the central area, with their noses facing one of the closed arms, and explored for 5 min. The time spent by the mice and rats exploring each area was recorded via camera and software.

**Marble burying test**

The marble burying test is used to assess the anxiety-like behavior. 20 standard glass beads (14 mm-15 mm in diameter) were arranged in a bedding-lined cage (4×5). The mice were gently placed in the corner of the cage and tested for 30 min. A glass bead was considered buried if more than two-thirds of it was covered by the bedding. The number of those was recorded.

**Y-maze test**

Y-maze, used to assess the memory ability of rodents, consists of 3 arms (for mice: 35×5×15 cm; for rats: 50×10×20 cm), the starting arm, the old arm and the new arm. It was divided into two periods, the training period and the testing period. During the training period, the new arm was blocked by the spacer and the mice were placed by the starting arm and moved freely in the starting arm and the old arm for 5 min. After the training, the mice were put back into the feeding cage. 1 h later, the test was performed by removing the spacer and the mouse was placed in the starting arm, moving freely in the 3 arms for 5 min. The camera and software were used to record the time spent exploring each arm.

**Novel object recognition (NOR)**

NOR is a non-rewarding experimental model used to assess the recognitive capability of rodents, also divided into a training period and a test period. During the training period, two identical objects were placed in the open field (ensuring that the objects were odorless and not pushed), 10 cm from either wall, and the mice were placed in the apparatus with their backs facing the objects from an equal distance and are free to explore for 5 min. The test was carried out after 1h. One of the objects was replaced with a different object and placed in the device, again with the mouse's back facing the object from an equal distance from it for 5 min. 1h later, the test phase is entered and one of the objects is replaced with a different object and placed in the apparatus, placing the mice in the device for 5 min as in the training period. The software was used to record the time spent exploring each object (touching the object with the mouth or nose and approaching the object within about 2-3 cm of the object) and to record the times of explorations.

**Tail suspension test (TST)**

The TST is used to reflect depression-like behavior in rodents. The tail of the mouse was fixed, specifically with medical tape 1cm from the tip of the tail, at the height of approximately 30 cm between the tail and the ground, so that the mouse was in a head-down position for 6 min. The camera recorded the entire procedure and the immobility time during the last 4 min was used as an indicator.

**Sucrose preference test (SPT)**

The SPT was used to assess anhedonia. In short, two water bottles containing 1% sucrose water were placed in each cage for 24 h. A water bottle containing 1% sucrose water and a water bottle containing pure water were randomly placed in the cage, and the location of the water bottle was changed after 12 h. Before the experiment, the rats were deprived of water and food for 24 h. The consumption of sucrose water and pure water in 24 h was recorded, and the sucrose preference index (SPI) was calculated: SPI = [sucrose water intake/ (sucrose water intake + pure water intake)] × 100%.

**Table S1: The list of primers used in RT-qPCR for mouse and astrocytes.**

| **Gene** | **Primer sequences (Forward)** | **Primer sequences (Reverse)** |
| --- | --- | --- |
| Slc2a1 | ATAGTTACAGCGCGTCCGTT | ATAGTTACAGCGCGTCCGTT |
| Slc2a3 | AACCAGGACTGCTTCTGAGTG | TTACAAGGAAGTATCCCCAAATCAA |
| Slc16a1 | GGGCTAAAGCCACAGTCCAT | TCTGCTAAGTGCCACACAGG |
| Slc16a7 | TCTGTAGCCTTGACCTCCTTGT | TAATGCCGTCTGTGTTCCCC |
| Slc16a3 | GTACCCATACCCGGCGTTAG | CCCCGTGTGTAAAGGTGTCA |
| Ldha | TGTGAGCCTGCTGCATTCG | TGCATCATGGACGTACACAC |
| Ldhb | GGATTCACCCCGTGTCTACC | GAGCGACCTCATCGTCCTTC |
| Hif1a | TGAGTTCTGAACGTCGAAAAGA | GGGGAAGTGGCAACTGATGA |
| Syp | GGGCCAATGATGGACTTCCT | GCCTGTCTCCTTGAACACGA |
| Dlg4 | AGTTGCAGGTGAACGGAACA | TCACCGATGTGTGGGTTGTC |
| Ppargc1a | GTGTTCTGGTACCCAAGGCA | ATGGTCACCAAACAGCCGAA |
| Sirt1 | CGGCTACCGAGGTCCATATAC | ACAATCTGCCACAGCGTCAT |
| Bdnf | CAGGACAGCAAAGCCACAAT | GCCTTCATGCAACCGAAGTA |
| Actb | CCCTGGACTTCGAGCAAGAG | GGCTGTATTCCCCTCCATCG |

Slc2a1, solute carrier family 2, member 1, also Glut-1; Slc2a3, solute carrier family 2, member 3 also Glut-3; Slc16a1, solute carrier family 16, member 1, also Mct1; Slc16a7, solute carrier family 16, member 7, also Mct2; Slc16a3, solute carrier family 16, member 3, also Mct4; Ldha, lactate dehydrogenase A; Ldhb, lactate dehydrogenase B; Hif1a, hypoxia inducible factor 1, alpha subunit; Syp, synaptophysin; Dlg4, discs large MAGUK scaffold protein 4, also PSD95; Ppargc1a, peroxisome proliferative activated receptor, gamma, coactivator 1 alpha, also Pgc-1alpha; Sirt1, sirtuin 1; Bdnf, brain derived neurotrophic factor; Actb, actin, beta

**Table S2: The list of primers used in RT-qPCR for rat.**

| **Gene** | **Primer sequences (Forward)** | **Primer sequences (Reverse)** |
| --- | --- | --- |
| Slc2a1 | GACTCCCAAGTGTGAGGAGC | ACTGAAGAAAGGTGCCCAGG |
| Slc2a3 | CCATCTCTGGTGTTCGCTGT | GTCTTCCAACCGCTCTTCCA |
| Slc16a1 | CACGAGCTGCGAAGTGACTG | CGGGCCGCAATTCTTCGG |
| Slc16a7 | TGCAGTCATCCAGTGTGGTT | AGAGCTGTGTGGGGGTTTAG |
| Slc16a3 | GGGGCACTGATGACACTACC | GCCCGTGATGATGAAACAGC |
| Ldha | GAGCTGTGGTTGGTCCAGTT | GCAGTTGGCAGTGTGTCTTG |
| Ldhb | TTGTCTGGACAAGATGGCAAC | GCCAGAGACTTCCCCAGAATG |
| Hif1a | CTTTCTCTGCGCGTGAGGAC | TCGACGTTCGGAACTCATCC |
| Syp | TCCTGTACCCTCTGCTGTGT | GCACAGGAAAGTAGGGGGTC |
| Dlg4 | TACCGCTACCAAGATGAAGACA | GTTCCATTCACCTGCAACTCA |
| Actb | GCGAGTACAACCTTCTTGCAGC | TGCCGGAGCCGTTGTCG |

Slc2a1, solute carrier family 2, member 1, also Glut1; Slc2a3, solute carrier family 2, member 3 also GLUT3; Slc16a1, solute carrier family 16, member 1, also MCT1; Slc16a7, solute carrier family 16, member 7, also Mct2; Slc16a3, solute carrier family 16, member 3, also MCT4; Ldha, lactate dehydrogenase A; Ldhb, lactate dehydrogenase B; Hif1a, hypoxia inducible factor 1 subunit alpht; Syp, synaptophysin; Dlg4, discs large MAGUK scaffold protein 4, also PSD95; Ppargc1a, peroxisome proliferative activated receptor, gamma, coactivator 1 alpha, also Pgc-1alpha; Sirt1, sirtuin 1; Bdnf, brain derived neurotrophic factor; Actb, actin, beta


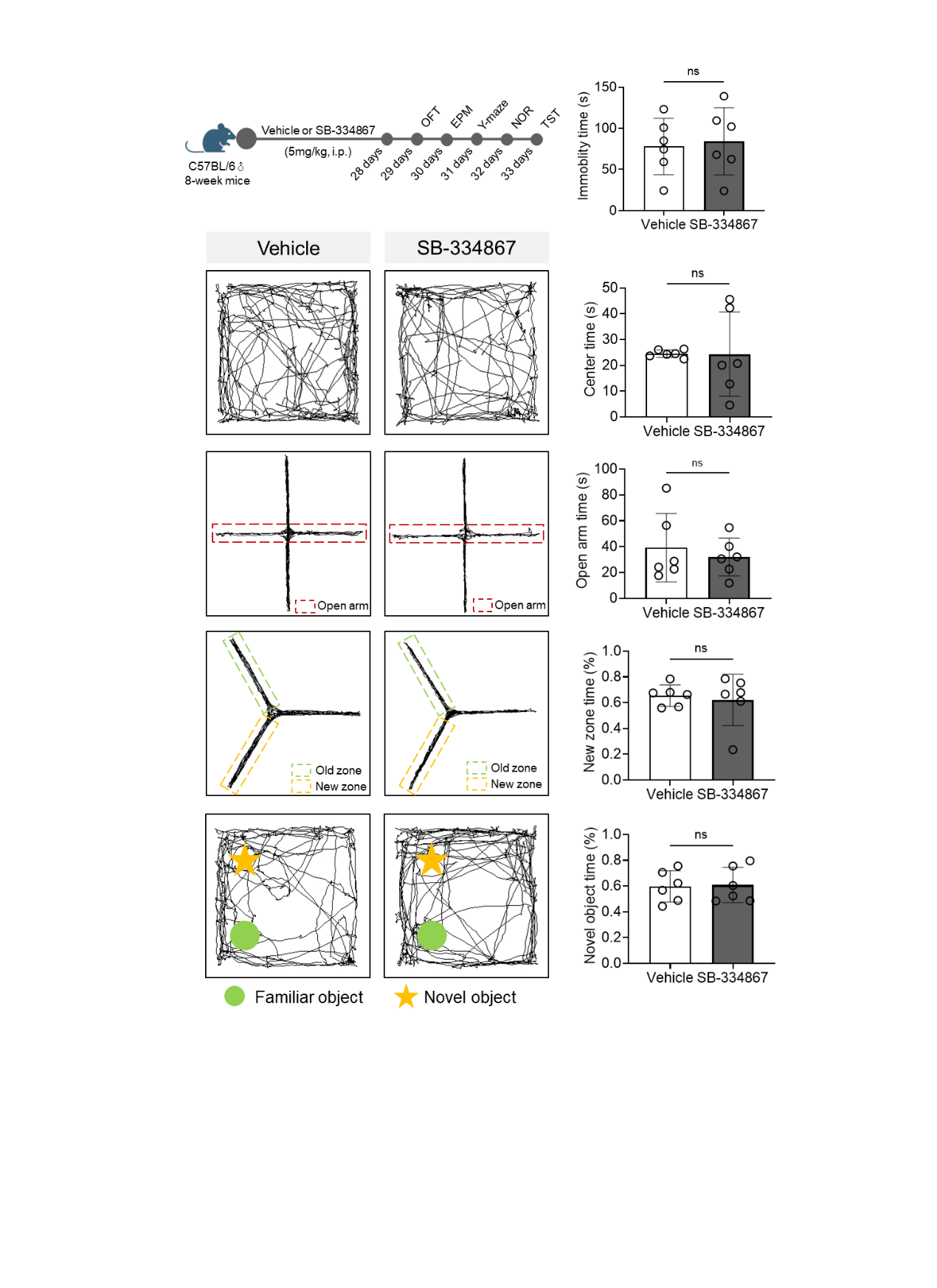


**Figure S1: The effects of SB-334867 on the behaviors of controls.** There was no significant difference between Vehicle and SB-334867 group in exploration time in the center in open field test and open arm in elevated plus maze, and the proportion of new zone and novel object time in novel object recognition and Y-maze, respectively, and the immobility time in tail suspension test.


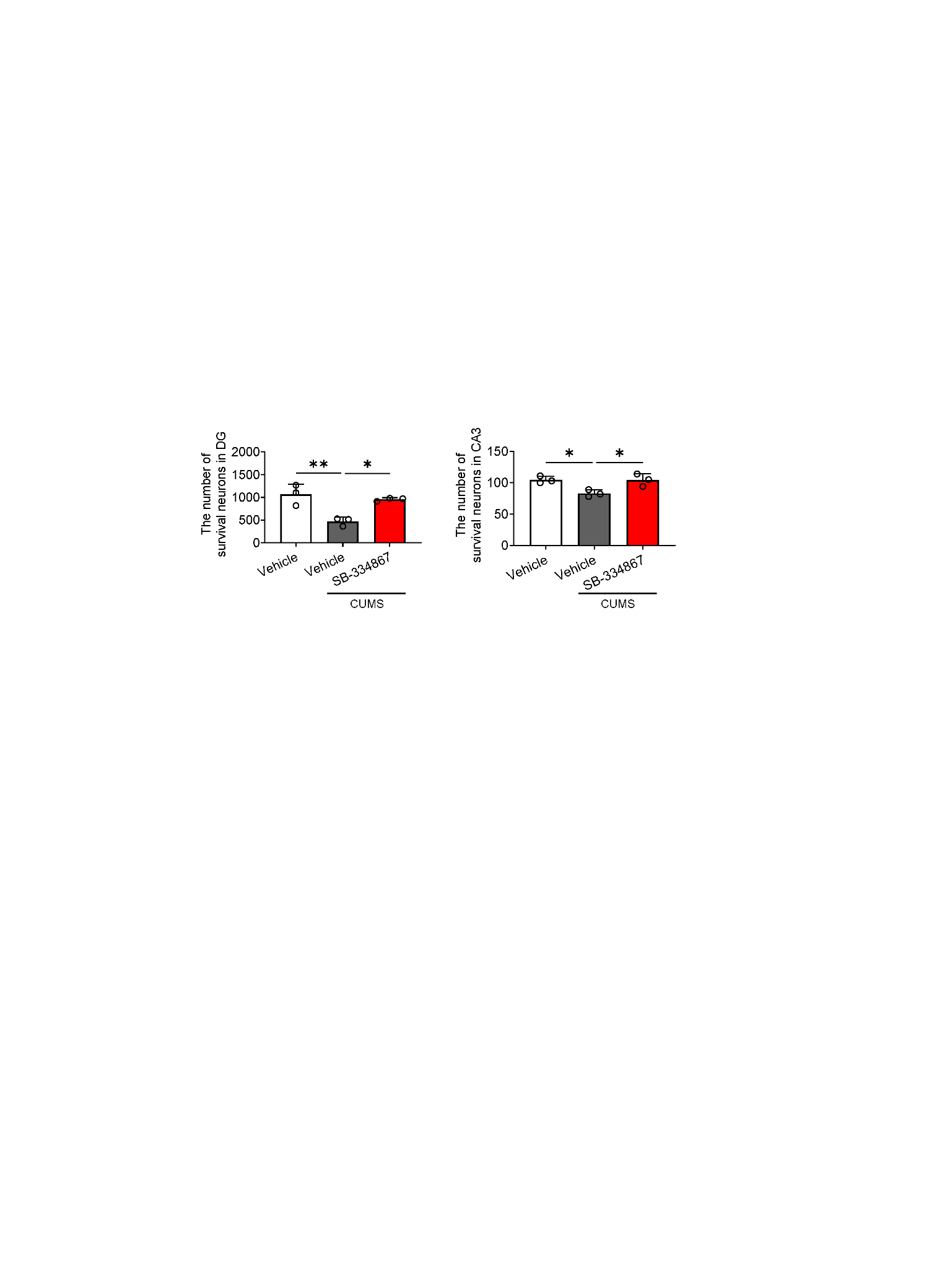


**Figure S2: Quantification of Nissl staining.** HCRTR1 antagonist reduced CUMS-induced neuronal damage in hippocampal CA3 and DG.


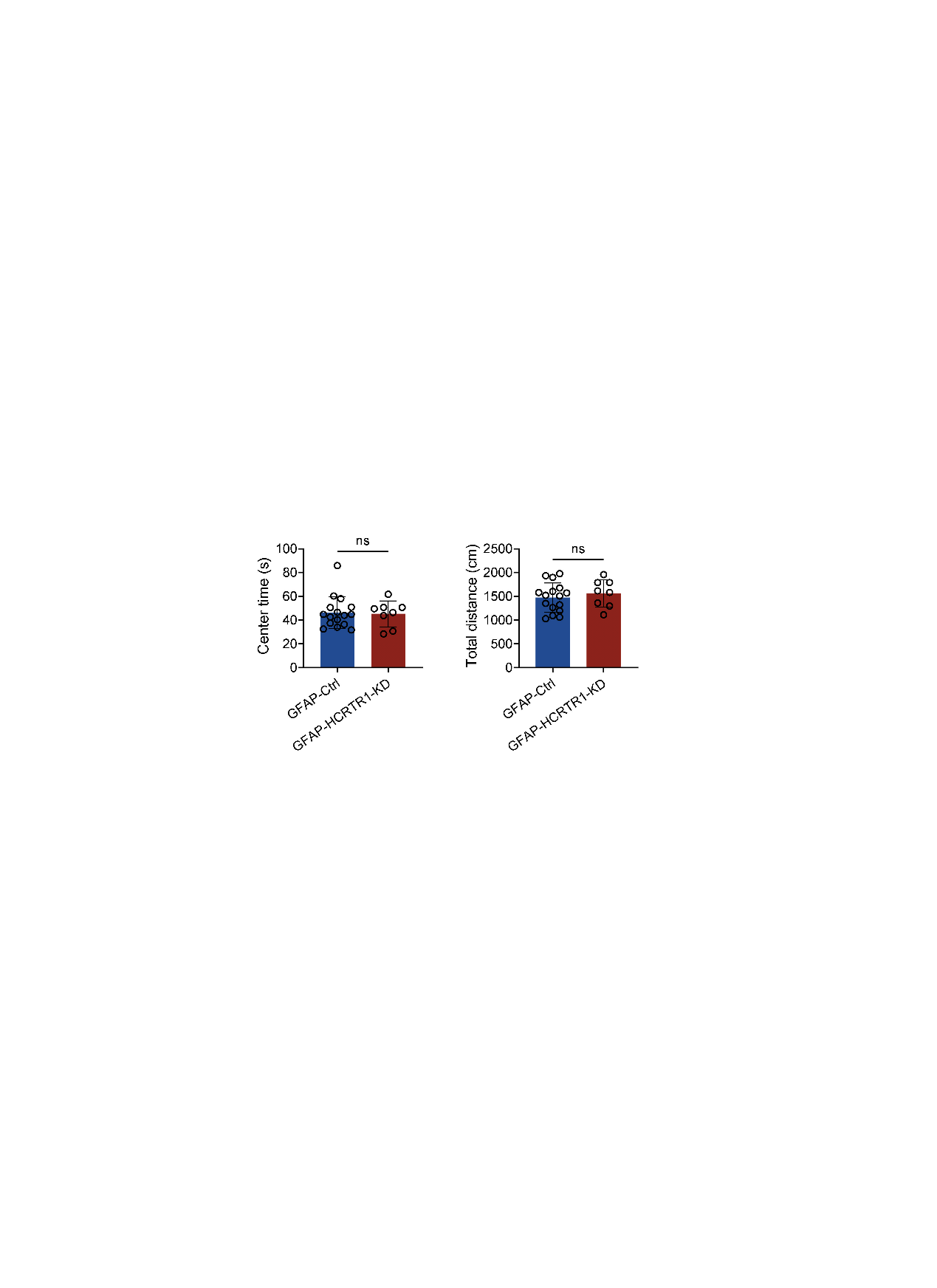


**Figure S3:** **The center time and total distance in open field test before CUMS procedure.** There was no significant difference between in GFAP-Ctrl and GFAP-HCRTR1-KD groups.


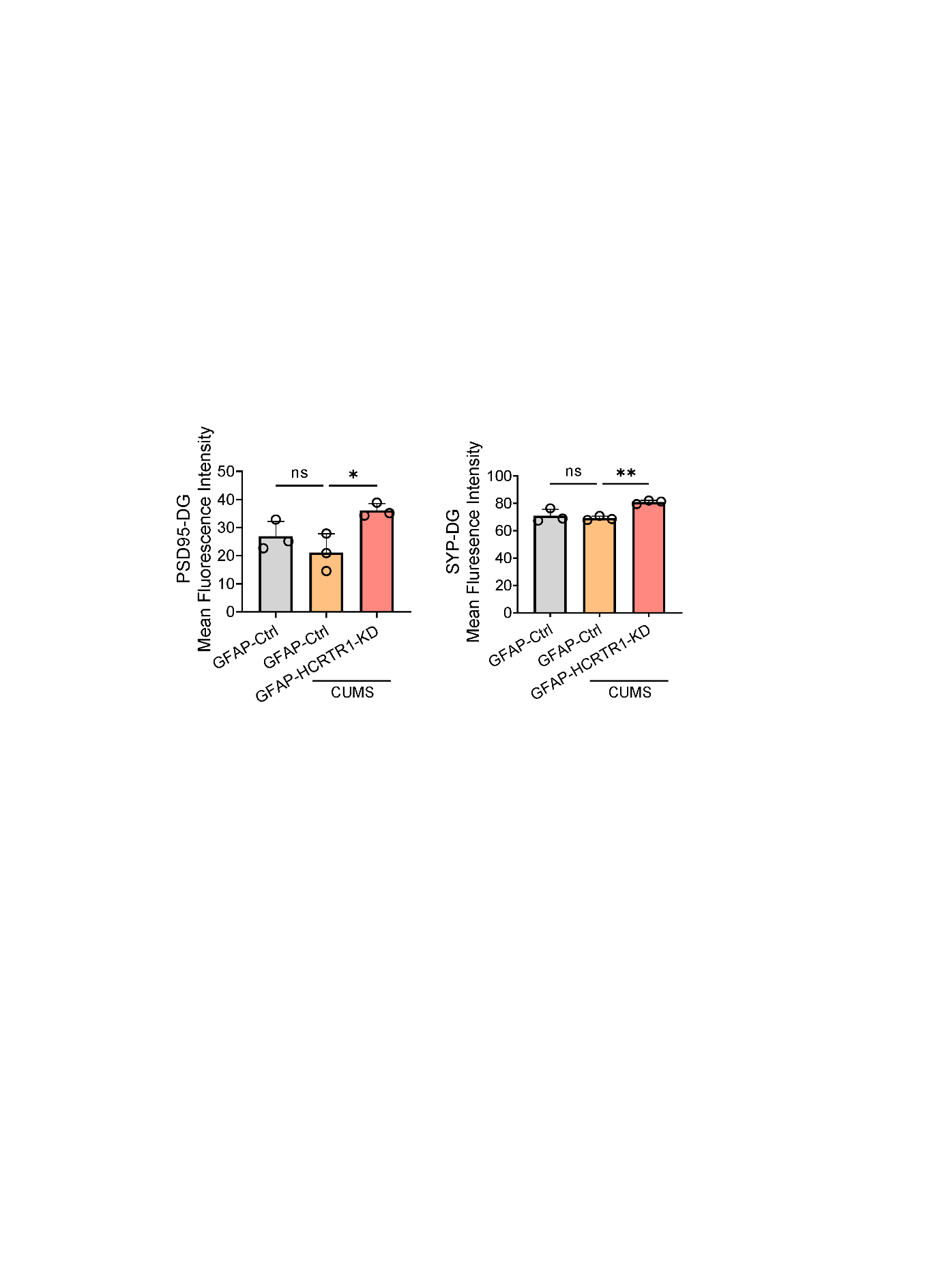


**Figure S4:** **Quantification of mean fluorescence intensity of PSD-95 and SYP in DG in different groups.**


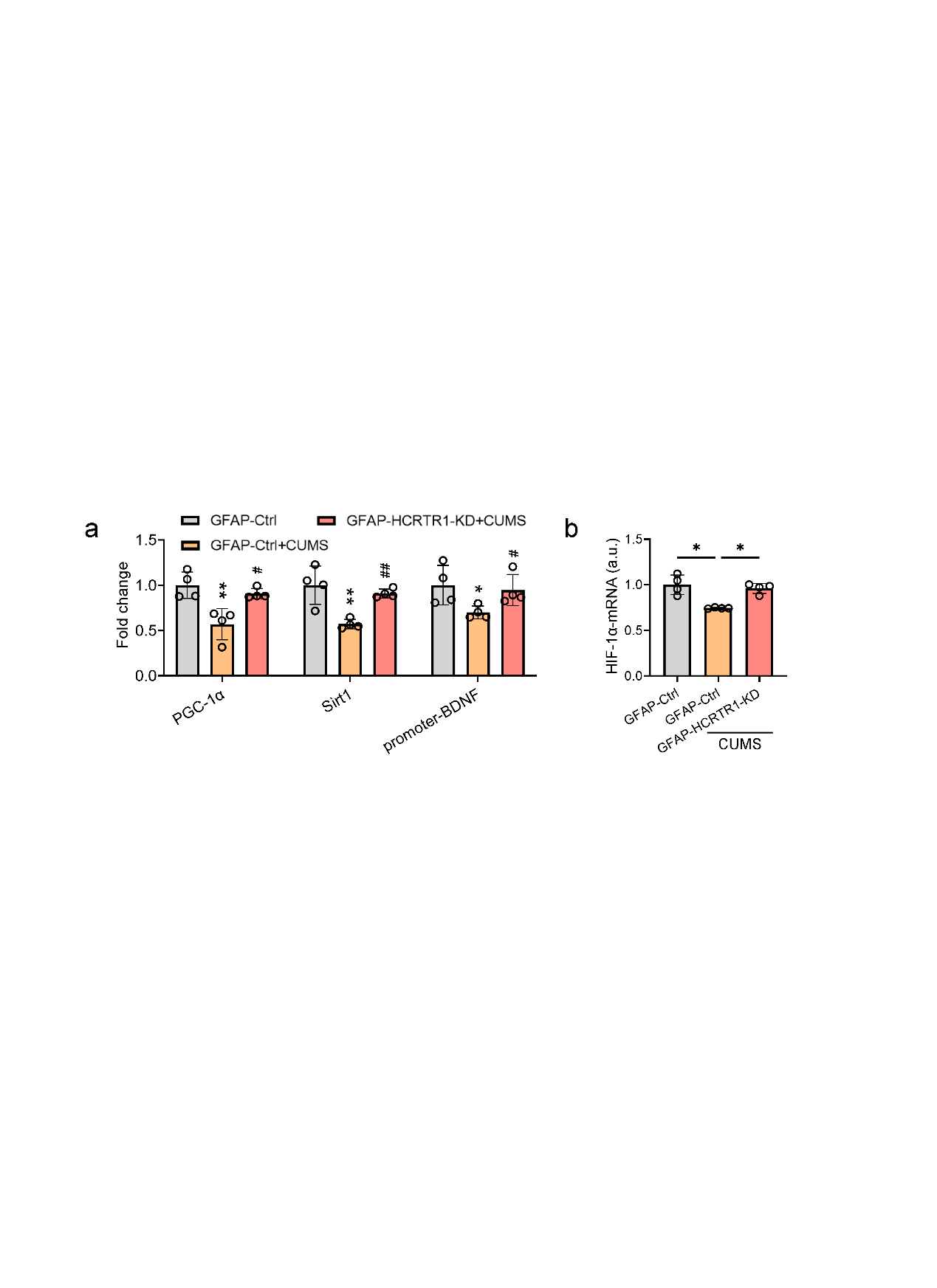


**Figure S5:** **The mRNA expression of PGC1α-Sirt1-BDNF pathway and HIF-1α in different groups.** Compared to GFAP-Ctrl group, the mRNA expression of PGC-1α, Sirt1 and promoter-BDNF and HIF-1α were significantly decreased in GFAP-Ctrl+CUMS group, which were significantly increased in GFAP-HCRTR1-KD group.


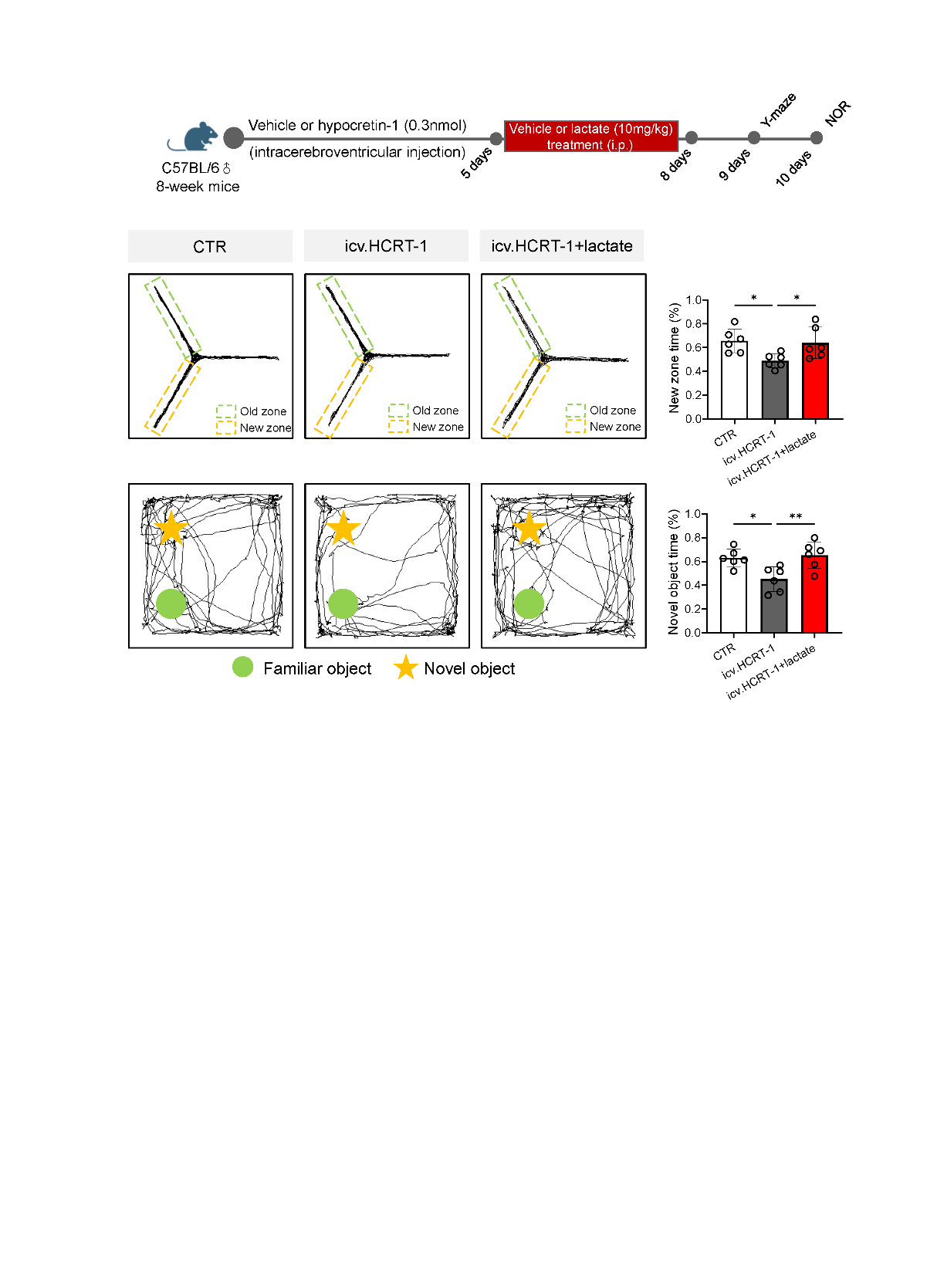


**Figure S6:** **Lactate could significantly ameliorate the cognitive impairment induced by hypocretin-1.** icv.HCRT-1 showed a decreased ratio of new zone and new object exploration time in the Y-maze and NOR, respectively, compared with the CTR group, while icv.HCRT-1+lactate significantly reversed.
